# Human oncostatin M deficiency underlies an inherited severe bone marrow failure syndrome

**DOI:** 10.1172/JCI180981

**Published:** 2025-01-23

**Authors:** Alexandrine Garrigue, Laëtitia Kermasson, Sandrine Susini, Ingrid Fert, Christopher B. Mahony, Hanem Sadek, Sonia Luce, Myriam Chouteau, Marina Cavazzana, Emmanuelle Six, Marie-Caroline Le Bousse-Kerdilès, Adrienne Anginot, Jean-Baptiste Souraud, Valérie Cormier-Daire, Marjolaine Willems, Anne Sirvent, Jennifer Russello, Isabelle Callebaut, Isabelle André, Julien Y. Bertrand, Chantal Lagresle-Peyrou, Patrick Revy

**Affiliations:** 1 Institut Imagine, Université Paris Cité, INSERM UMR1163, Laboratory of Human Lymphohematopoiesis, Paris, France.; 2 INSERM UMR 1163, Laboratory of Genome Dynamics in the Immune System, Équipe Labellisée LIGUE 2023, Paris, France.; 3 Paris Cité University, Imagine Institute, Paris, France.; 4 University of Geneva, Faculty of Medicine, Department of Pathology and Immunology, Geneva, Switzerland; Geneva Centre for Inflammation Research, Faculty of Medicine, University of Geneva, Geneva, Switzerland.; 5 Service de Biothérapie et d’Aphérèse, Hôpital Necker, Groupe Hospitalier Universitaire Ouest, Assistance Publique-Hôpitaux de Paris (AP-HP), Paris, France.; 6 INSERM UMRS-MD 1197, Université de Paris-Saclay, Hôpital Paul Brousse, Villejuif, France.; 7 Service Anatomo-Pathologie, Hôpital d’Instruction des Armées Begin, Saint-Mandé, France.; 8 Reference Center for Skeletal Dysplasia, Hôpital Necker-Enfants Malades, Assistance Publique-Hôpitaux de Paris, Paris, France.; 9 Medical Genetics Department, CHU de Montpellier, Montpellier, France.; 10 Department of Pediatric Oncology and Haematology, Montpellier Hospital, Montpellier, France.; 11 Service d’Hématologie Biologique, CHU de Montpellier, Montpellier, France.; 12 Sorbonne Université, Muséum National d’Histoire Naturelle, UMR CNRS 7590, Institut de Minéralogie, de Physique des Matériaux et de Cosmochimie, IMPMC, Paris 75005, France.; 13 Centre d’Investigation Clinique Biothérapie, Groupe Hospitalier Universitaire Ouest, AP-HP, Paris, France.

**Keywords:** Genetics, Hematology, Bone marrow, Cytokines, Hematopoietic stem cells

## Abstract

Oncostatin M (OSM) is a cytokine with the unique ability to interact with both the OSM receptor (OSMR) and the leukemia inhibitory factor receptor (LIFR). On the other hand, OSMR interacts with IL31RA to form the interleukin-31 receptor. This intricate network of cytokines and receptors makes it difficult to understand the specific function of OSM. While monoallelic loss-of-function (LoF) mutations in OSMR underlie autosomal dominant familial primary localized cutaneous amyloidosis, the in vivo consequences of human OSM deficiency have never been reported so far. Here, we identified 3 young individuals from a consanguineous family presenting with inherited severe bone marrow failure syndromes (IBMFS) characterized by profound anemia, thrombocytopenia, and neutropenia. Genetic analysis revealed a homozygous 1 base-pair insertion in the sequence of *OSM* associated with the disease. Structural and functional analyses showed that this variant causes a frameshift that replaces the C-terminal portion of OSM, which contains the FxxK motif that interacts with both OSMR and LIFR, with a neopeptide. The lack of detection and signaling of the mutant OSM suggests a LoF mutation. Analysis of zebrafish models further supported the role of the OSM/OSMR signaling in erythroid progenitor proliferation and neutrophil differentiation. Our study provides the previously uncharacterized and unexpectedly limited in vivo consequence of OSM deficiency in humans.

## Abstract 2

Human *OSM* homozygous loss-of-function variants cause profound anemia, thrombocytopenia and neutropenia Human OSM-deficient hematopoietic stem and progenitor cells can differentiate into erythroblasts and megakaryocytes

## Graphical abstract

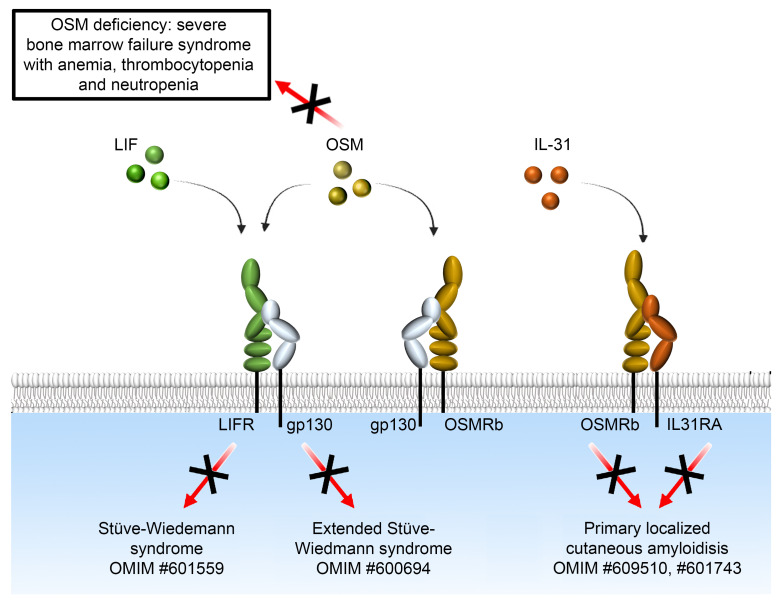

## Introduction

Inherited severe bone marrow failure syndromes (IBMFS) represent a group of life-threatening rare genetic disorders sharing a common feature of impaired production of one or more blood cell lineages (1). IBMFS can be associated with other clinical manifestations, including growth retardation, mucocutaneous abnormalities, developmental defects, and susceptibility to develop myelodysplastic syndrome and cancer. The identification of various genes associated with IBMFS has facilitated the categorization into distinct main groups. One of these encompasses ribosomopathies, specifically Diamond-Blackfan anemia (DBA) and Shwachman-Diamond syndrome (SDS), which result from genetic defects affecting ribosome production and maturation (2, 3). A second group comprises disorders linked to genetic defects in factors participating in DNA repair and chromosomal stability. While this group encompasses several distinct disorders (4, 5), a prototypical example is Fanconi anemia (FA), a DNA repair syndrome involving 22 identified causative genes. Another group pertains to telomere biology disorders, including Dyskeratosis congenita and its severe variant, Høyeraal-Hreidarsson syndrome, caused by genetic defects impacting telomere integrity (6, 7). Finally, the other IBMFS entities include SAMD9/9L syndromes, severe congenital neutropenia (SCN), and congenital thrombocytopenia and anemia, which can arise from mutations in genes involved in erythro/myeloid differentiation, regulation of metabolism, or hematopoietic cell homeostasis (1, 8, 9).

Oncostatin M (OSM) belongs to the interleukin-6 group of cytokines (10). Of these cytokines, OSM most closely resembles leukemia inhibitory factor (LIF) in both structure and function. Primary structure analysis of OSM allocates it to the gp130 group of cytokines. As yet poorly defined, OSM appears to play pleiotropic roles, including bone formation and destruction (11), liver development, hematopoiesis, inflammation regulation, wound healing, and CNS development (12, 13). The full human OSM (hOSM) cDNA encodes a 252 amino acid precursor, the first 25 amino acids of which function as a secretory signal peptide, which on removal yield the soluble 227 amino acids pro-OSM. Cleavage of the last 31 residues at the C-terminal part at a trypsin-like cleavage site gives rise to the fully active OSM form containing 196 residues. Two potential N-glycosylation sites are present in hOSM, both of which are retained in the mature form (14, 15). OSM shares 22% sequence identity with LIF. Noticeably, *OSM* and *LIF* genes locate in tandem on human chromosome 22 and exhibit similar promoter elements and intron-exon structure (16) suggesting that they arose by gene duplication relatively recently during evolution (17). OSM is particularly expressed by activated macrophages, monocytes, T cells, neutrophils, and dendritic cells (10, 18). In humans, there are 2 distinct categories of functional OSM receptors (OSMRs). The first category, known as the type I OSM receptor, corresponds to the high-affinity LIF receptor (LIFR), composed of gp130 and the LIF receptor subunit. The second category, termed type II OSM receptor, comprises gp130 and the OSM-specific receptor subunit (OSMR). On the other hand, OSMR constitutes, together with the IL-31 receptor subunit (IL31RA), the receptor of the IL-31 cytokine (13). In mice, Osm deficiency results in the massive accumulation of apoptotic thymocytes and production of autoantibodies, suggesting that Osm plays a key role in the prevention of autoimmune disease by regulating the clearance of apoptotic thymocytes (19). Conversely, transgenic mice that express Osm under the lck proximal promoter exhibit thymic hypertrophy (20, 21). Besides that effect on thymic function, Osm-deficient mice exhibit several hematopoietic abnormalities. Hematopoietic progenitor activity was shown to be reduced in the bone marrow while increased in the spleen and peripheral blood, correlating with an increased level of circulating granulocyte colony-stimulating factor (G-CSF) and a decreased level of CXCL12 (also known as stromal cell-derived factor 1 [SDF-1]) (22). Mutant mice deficient in the Osm-specific receptor β subunit (Osmr) had slight but significant reduced numbers of peripheral erythrocytes and platelets and reduced numbers of erythroid and megakaryocyte progenitors in the bone marrow (23). Transplantation experiments showed that Osm regulates hematopoiesis in vivo by stimulating stromal cells as well as hematopoietic progenitors, in particular megakaryocytic and erythrocytic progenitors. Thus, while studies conducted in murine and cellular models demonstrated pleiotropic functions of OSM, the in vivo consequences of OSM deficiency in humans remained unknown.

We report here 3 individuals with profound anemia, thrombocytopenia, and neutropenia caused by a homozygous LoF mutation in OSM.

## Results

### Patients’ biological features.

**Figure 1:**
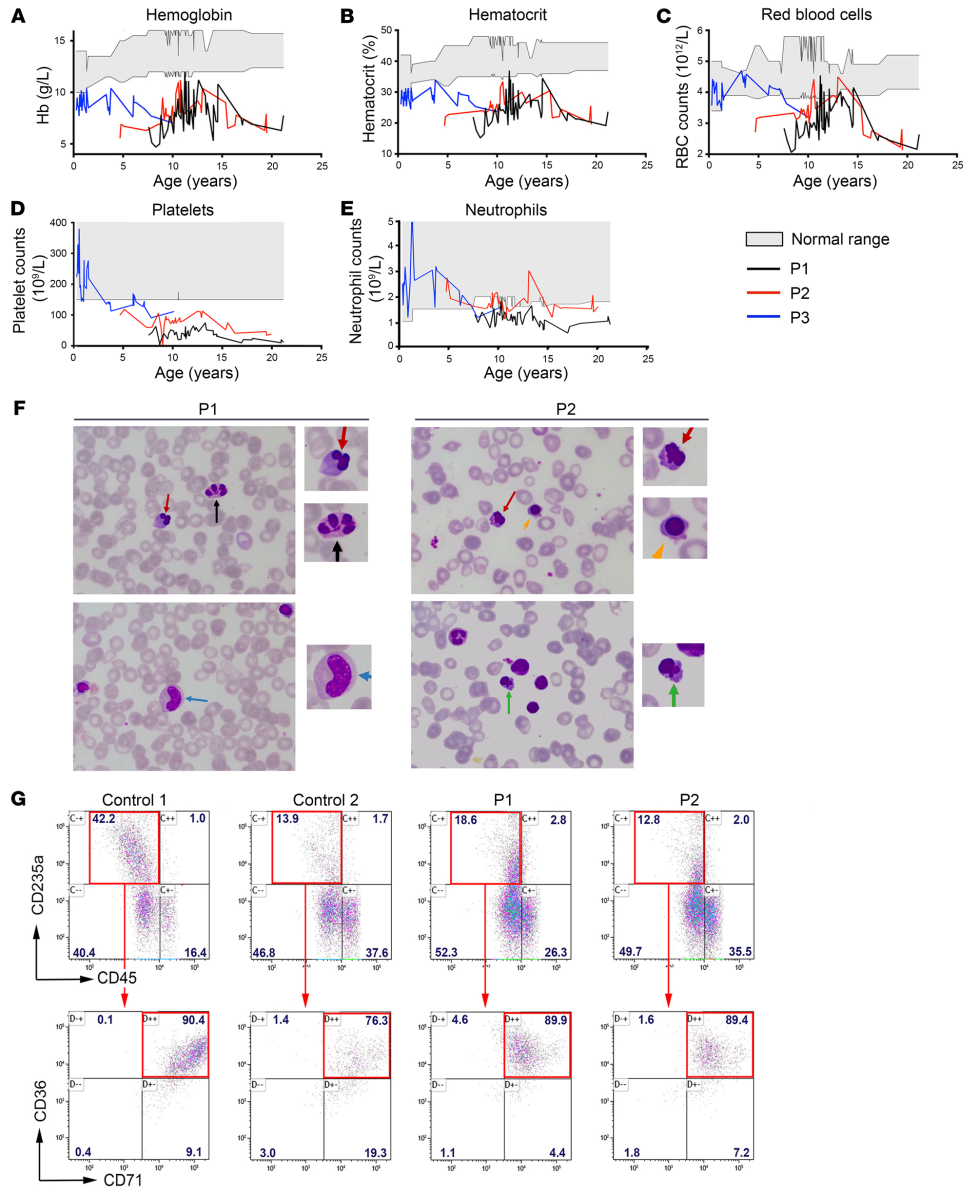
Evolution of hematologic values in the 3 patients, cytological analysis of bone marrow smears, and in vitro erythrocyte differentiation. Variation of hemoglobin (g/dL) (**A**), hematocrit (%) (**B**), RBC (**C**), platelets (**D**), and neutrophil counts (**E**), in P1, P2, and P3 according to age. Gray zone indicates normal ranges in healthy individuals. The RBC counts were low in patients, even after RBC transfusion. (**F**) Cytological findings of bone marrow smears in P1 and P2. Irregular budding nucleus (red arrow), internuclear bridges (black arrow), acidophilic erythroblasts (green arrow), and polychromatophilic erythroblasts (orange arrow) were detected. Dysgranulopoiesis was observed in P1 as several metamyelocytes did not contain any granules (blue arrow). The slides were stained with Wright-Giemsa and H&E. Original magnification, ×100; ×175 (zoomed images). Abnormal cells are zoomed-in on the right. (**G**) In vitro differentiation of HSPCs into erythroblasts. Top: CD235a/CD45 staining after 7 days of culture in erythroid conditions of HSPCs from 2 healthy controls and patients P1 and P2. The red rectangle indicates the gating strategy on CD235a^+^CD45^–^ erythroid cells. Bottom; CD36/CD71 staining of CD235a^+^CD45^–^ cells. The red rectangle indicates CD36^+^CD71^+^ erythroblast cells.

The proband II. 2 (hereafter noted P1) was referred to hospital at the age 6 months for profound anemia (Figure 1, A–C), thrombocytopenia (Figure 1D), and fluctuant mild neutropenia (Figure 1E and Table 1 for the last follow-up). She belongs to a consanguineous Moroccan family of 5 children, 1 male, 4 females (Supplemental Figure 1A; supplemental material available online with this article; https://doi.org/10.1172/JCI180981DS1). Two of her sisters (II.3 and II.5, hereafter noted P2 and P3, respectively) presented with anemia between 3 and 6 months of age. At 4 years of age, P2 also exhibited thrombocytopenia (Figure 1 and Table 1). At the last follow-up of their blood count, hemoglobin (61 to 77 g/L) and hematocrit (19.7% to 23.6%) values were low in the 3 patients as were RBC counts (between 2.15 and 3.3 × 10^12^/L) (Figure 1 and Table 1). Mean corpuscular volumes, mean corpuscular hemoglobin, and mean corpuscular hemoglobin concentrations were in the normal ranges (Table 1). The number of reticulocytes was comparable in patients and in aged-matched controls. Conversely, erythroblasts were found at high proportions (18% in P1 and 15% in P2) and red cell distribution width was higher than normal (21.3% to 26.2%) while signs of iron deficiency or hemolysis were absent (data not shown). Haptoglobin level was measured twice in P1 at 11 years of age and was found normal (data not shown). Taken together, hematological features of the 3 patients were evocative of an aregenerative normocytic normochromic anemia suggesting an impaired RBC production. The thrombocytopenia, present in all 3 patients, was particularly pronounced in P1 (Figure 1D and Table 1). Remarkably, although platelet counts were normal during the first 3 years of life of P3, we noted a progressive decrease in these values to reach levels below the normal (Figure 1D and Table 1). In addition, P1 and P2 had consistently low neutrophil counts (Figure 1E), whereas neutrophil counts were in the normal range for the first 6 years of P3’s life, but progressively decreased to abnormally low levels (Figure 1E and Table 1). The results of the osteo-medullary biopsies and myelogram in P1 indicated a cell density that was normal up to 15 years of age, but became low at 18 years of age (Supplemental Table 1). The granulocyte ratio was decreased, the lymphoid ratio was increased, and the erythroid ratio was in the normal range, but decreased with age. The myeloid/erythroid ratio was low but increased with age (Supplemental Table 1). Cytological analysis of bone marrow samples (P1 at the age of 9.5 and P2 at the age of 7) revealed a mild to moderate dyserythropoiesis with irregular budding nucleus or internuclear bridges (Figure 1F). For P1, the detection of hypogranular and dystrophic precursors was suggestive of dysgranulopoiesis (Figure 1F). Of note, megakaryocytes were not numerous enough to be studied. Acidophilic and polychromatophilic erythroblasts (P2) were also observed (Figure 1F). The absence of increased reticulocytes in the peripheral blood suggested the presence of an aregenerative anemia, characterized by a maturation blockage at the erythroblastic stage. We also noted the presence of 2% to 6% of circulating myelocytes usually associated with neutropenia, an observation in accordance with an ineffective granulopoiesis. Finally, the platelets were lowered with a subnormal (7%) immature platelet fraction (IPF), which accounts for a defective central platelet production.

Furthermore, when comparing the number of hematopoietic stem and progenitor cells (HSPCs) using CD34^+^ and CD45^+^ surface antigen in peripheral blood mononuclear cells (PBMNC), we did not observe an increase in percentage of circulating HSPCs in the 3 patients (0.13%, 0.04%, and 0.18% for P1, P2, and P3, respectively) compared with the healthy brother (0.34%), a healthy control (0.71%) and the normal range (0.01–1.3%) according to ref. 24.

All other blood lineages (lymphocytes, monocytes, eosinophils, and basophils) were within the normal ranges (Table 1). Impairment of erythroid, platelet and neutrophil lineages strongly suggested a central bone marrow failure, particularly severe for the older patient, P1.

Anemia was treated by regular erythrocyte transfusions, the frequencies of which increased over time (currently every 3 weeks for P1, every 2 to 3 months for P2, and every 4 to 6 months for P3).

To determine whether the hematopoietic anomalies observed in patients originated from an intrinsic inability of HSPCs to differentiate, we performed colony-forming unit (CFU) assays. Bone marrow CD34^+^ HSPCs from P1 and P2 gave colony forming unit–granulocyte/macrophage (CFU-GM) and erythroid burst-forming units (BFU-E) counts within normal ranges (Supplemental Table 2). In vitro differentiation assay confirmed the capacity of bone marrow HSPCs from P1 and P2 to differentiate into erythroblasts, as inferred by the detection of CD235a^+^CD36^+^CD71^+^CD45^–^ cells (Figure 1G). Furthermore, in vitro mega-cult assays yielded a comparable proportion of megakaryocyte colonies in P1 and P2 patients and a healthy control (Supplemental Table 2). Taken together, these results suggest that the anemia and thrombocytopenia observed in the patients were caused either by a cell-intrinsic defect of HSPCs to differentiate beyond the erythroblast and megakaryocyte stages and/or by an alteration of the bone marrow environment.

Apart from the hematopoietic defects, all 3 patients were asymptomatic. Their growth was normal, with weight and height within the normal range. They showed no particular susceptibility to infection, nor any bone or psychomotor defects.

### Identification of homozygous OSM variant in patients.

**Figure 2:**
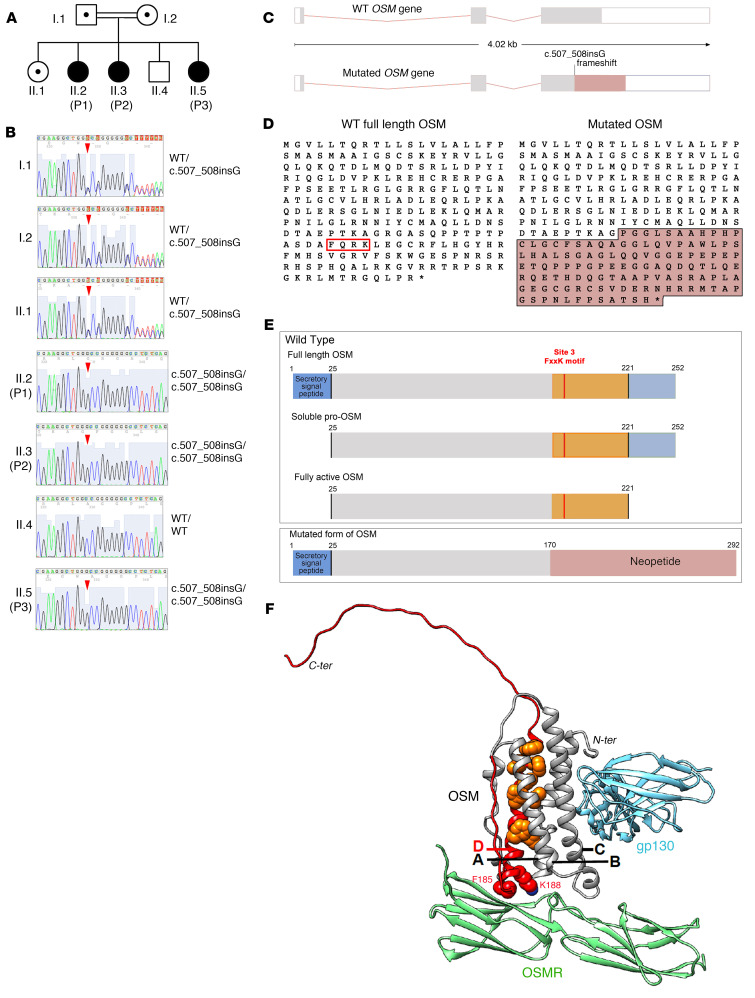
Identification of a homozygous OSM mutation in the patients. (**A**) Family pedigree. (**B**) Direct Sanger sequencing of the OSM gene in family members. Red arrowheads indicate the position of the 1 bp insertion causing a frameshift (c.507_508insG; Arg170AlafsTer124). (**C**) Schematic of the OSM gene showing the position of the frameshift leading to the production of a neo coding sequence (purple). (**D**) Protein sequences of the WT and mutated forms of OSM. Sequence in purple corresponds to the neopeptide generated by the frameshift. The red box highlights the FxxK motif critical to interact with OSMR and LIFR. (**E**) Domain architecture of the immature and mature forms of human OSM protein and the OSM mutated protein. Orange portion highlights the part that is missing in the mutated form of OSM and replaced by the neopeptide generated by the frameshift (in purple). Site 3 FxxK corresponds to the motif that interacts with OSMR and LIFR. (**F**) AlphaFold2 model of the 3D structure of WT human type II OSMR complex (OSM (UniProt P13725, aa 25-252) in complex with OSMR (UniProt Q99650, aa 141–330) and gp130 (UniProt P40189, aa 124–323). Details of the AF2 modeling are given in Supplemental Figure 6. The helices of the OSM 4-helix bundle are labeled. The phenylalanine and lysine of the FxxK motif are shown in a sphere representation (red) as well as hydrophobic amino acids of helix D (orange). The orange and red parts correspond to the missing domains in the OSMfs mutant created by the c.507_508insG variant.

In order to identify the molecular etiology of this bone marrow failure syndrome, we combined whole-genome homozygosity mapping (WGHM) in individuals I.1, I.2, II.1, P1, P2, and II.4 (Supplemental Figure 1) and whole-exome sequencing (WES) in P1 and P2. The consanguineous status of the family (Figure 2A) suggested an autosomal recessive inheritance of the disease and the presence of a pathogenic variant in the homozygous state only in the patients. WES analysis in P1 and P2 identified a homozygous 1 base-pair insertion in the coding sequence of OSM at position 507 (c.507_508insG; Chr22[GRCh37]: g.30660123_30660124insC). Importantly, the *OSM* gene is located in a region of chromosome 22 compatible with the WGHM analysis (Supplemental Figure 1). Sanger sequencing confirmed the presence of the variant in a homozygous state in the 3 patients (Figure 2B). Both parents and the healthy sister were heterozygous for the variant, while the healthy brother carried the WT allele in a homozygous state (Figure 2B). Notably, we also identified homozygous variants in the *FBLX17* and *DGKG* genes in the patients. However, several tools predicted a nondeleterious effect of these variants, which were also present in the gnomAD database (https://gnomad.broadinstitute.org/) (version 4 0.0; ref. 25) (Supplemental Figure 2). We thus considered the *OSM* variant as the strongest candidate since (a) it is absent from gnomAD, ClinVar (https://www.clinicalgenome.org/data-sharing/clinvar/), and LOVD databases (https://www.lovd.nl/) (Supplemental Table 3), (b) it cosegregates with the disease in the family, and (c) it affects the *OSM* gene, which is reported to participate in hematopoiesis (12, 26).

### Structural impact of the OSM mutation.

The c.507_508insG insertion in *OSM* causes a frameshift starting at codon Arg170 that generates a new reading frame predicted to create a neopeptide that ends 123 amino acids downstream (p.Arg170AlafsTer124, OSM mutant hereafter noted OSMfs) (Figure 2, C and D). Reverse transcriptase PCR (RT-PCR) amplification and sequencing analysis indicated that the full-length OSM mutant transcript was not subject to instability, degradation, or splicing aberrations (Supplemental Figure 3). This result suggested that this mutated transcript could theoretically be translated into OSMfs protein (Figure 2, D and E). Analysis of the 3D structure (AlphaFold2 model of the human OSM:OSMR:gp130 [type II OSMR] complex, details in Supplemental Figures 4 and 5) indicated that the putative OSMfs protein lacks the C-terminal portion of OSM (Figure 2F), which includes the fourth helix (D) from the 4-helix bundle fold typical of the hematopoietin cytokine family, while the neopeptide is predicted to be mainly disordered (Supplemental Figure 6). Importantly, OSMfs lacks the site 3 FxxK motif (F185-QR-K188, beginning of helix D) necessary to bind OSMR (Figures 2, D–F). This deletion also deprives the 4-helix bundle of hydrophobic amino acids in helix D (orange in Figure 2F and Supplemental Figure 6), essential for fold integrity. However, the neopeptide contains patches of hydrophobic amino acids similar to those found at the level of WT OSM helix D (Supplemental Figure 6). These amino acids occupy similar positions in the 3D structure models of the WT OSM and OSMfs, oriented toward the protein hydrophobic core (Supplemental Figure 6). Although modeled with low predicted local distance difference test (pLDDT) values, this fragment in OSMfs appears to be structurally coherent and could allow OSMfs to fold, likely in a less stable way, with potential indirect impact on the protein ability to interact with gp130 (blue in Figure 2F), via site 2. Taken together, these results suggest that the homozygous insertion c.507_508insG; p.Arg170AlafsTer124 found in patients disrupts OSM functional sites, while probably also having an effect on protein stability and represents a loss-of-function (LoF) mutation.

### Functional impact of the OSM mutation.

**Figure 3:**
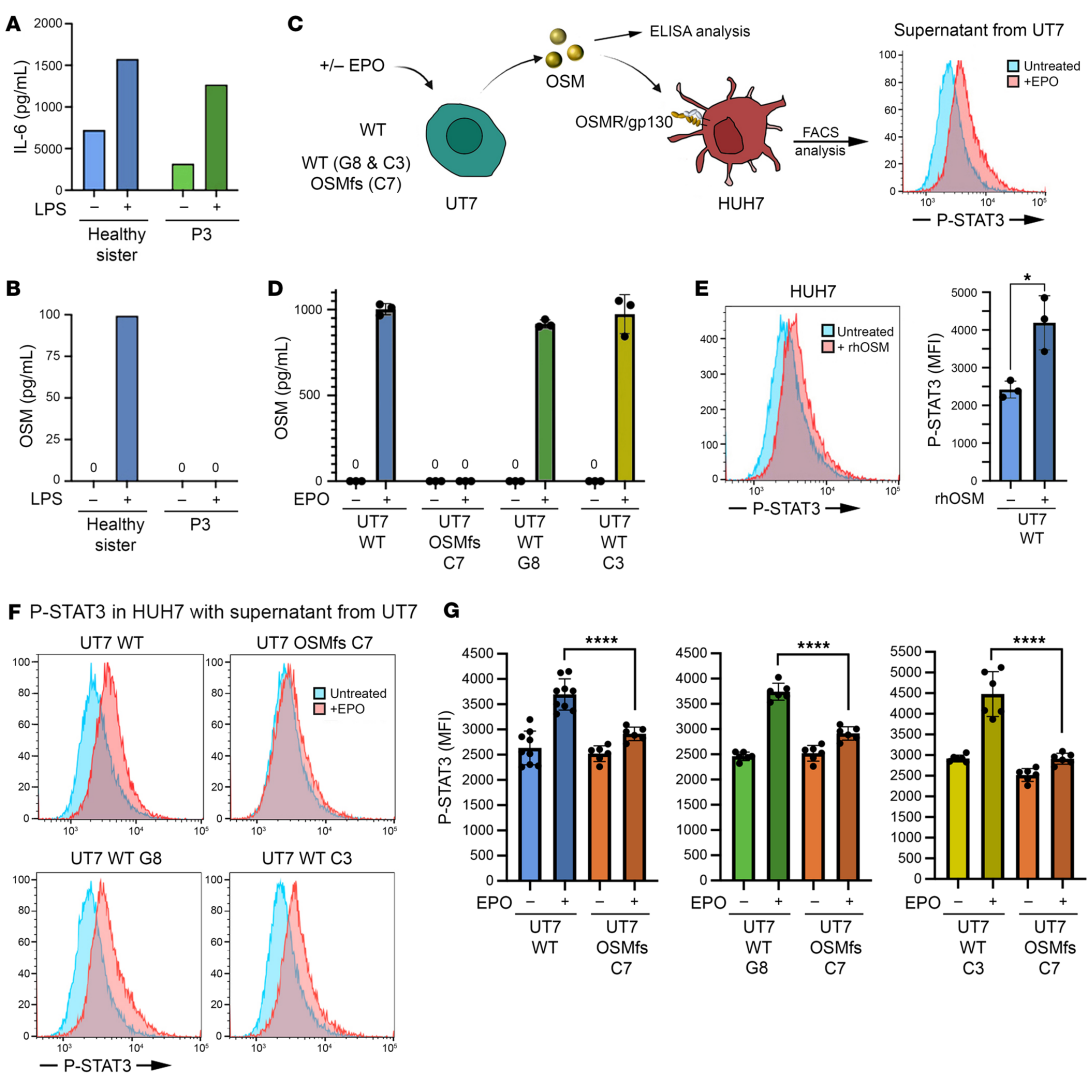
Functional consequence of the OSM mutation. (**A**) IL-6 concentration measured by ELISA in supernatants from LPS-activated monocytes from the healthy sister and P3. (**B**) OSM concentration measured by ELISA in supernatants from LPS-activated monocytes from the healthy sister and P3. (**C**) Schematic representation of the protocol used to measure OSM production by ELISA in supernatant from EPO-activated UT7 cells and its signaling detection in HUH7. OSM induces STAT3 phosphorylation (pSTAT3) in HUH7 as detected by FACS. (**D**) OSM concentration measured by ELISA in supernatants from EPO-activated WT UT7 cell line, the UT7 clone carrying the frameshift in the OSM sequence (clone C7), and 2 WT UT7 clones (C3 and G8). (**E**) Left: Representative FACS result demonstrating that rhOSM, used as positive control, induced a shift in STAT3 phosphorylation signal in HUH7 cells. Right: Quantitative analysis of 3 independent replicates. Statistics derived from unpaired *t* tests with α = 0.05. *P < 0.05. (**F**) Representative FACS results of p-STAT3 detection in HUH7 cells in the presence of indicated supernatants. (**G**) p-STAT3 induction obtained with the supernatants from EPO-activated WT UT7 cell line, the UT7 clone carrying the frameshift in the OSM sequence (clone C7), and 2 WT UT7 clones (C3 and G8). Quantitative analysis of 3 independent replicates. Statistics derived from unpaired *t* tests with α = 0.05. ****P < 0.0001.

To determine whether OSMfs could be detected in patient cells, we analyzed the supernatants of purified monocytes activated by LPS, which are expected to produce both IL-6 and OSM (27). The similar detection of IL-6 in supernatants from LPS-activated monocytes from P3 and her healthy sister II.1 (who carries the *OSM* mutation in a heterozygous state, Figure 2B) attested for an efficient activation of these cells (Figure 3A). However, while the ELISA detected OSM in the supernatant of LPS-activated monocytes from II.1, no signal was obtained in the supernatant of activated P3’s monocytes (Figure 3B). Based on these findings, we concluded that if OSMfs is produced and secreted by patients’ cells, its detection by ELISA cannot be achieved due to the marked structural modification generated by the frameshift. To further investigate the functional impact of the mutation in another setting, we introduced the *OSM* c.507_508insG insertion via CRISPR/Cas9 in the OSM-producing UT7 cell line. We successfully generated a clone (clone C7) with a homozygous 1 base-pair insertion that results in the same frameshift as seen in the patients (hereafter noted UT7^C7-OSMfs^; Supplemental Figure 7). We incubated the WT UT7 cell line, 2 UT7 clones that underwent CRISPR/Cas9 transfection but were WT for *OSM* (hereafter noted UT7^G8-WT^ and UT7^C3-WT^), and UT7^C7-OSMfs^ with erythropoietin (EPO) in order to induce OSM production (Figure 3C) (28). ELISA assay consistently detected OSM in the supernatants from EPO-activated WT UT7, UT7^G8-WT^, and UT7^C3-WT^ cells (Figure 3D), confirming their ability to produce OSM upon EPO treatment. In contrast, OSM was not detected in supernatant from the EPO-activated UT7^C7-OSMfs^ clone (Figure 3D), an observation consistent with the results obtained with patients’ cells (Figure 3B). However, the lack of detection of OSMfs does not rule out the possibility that OSMfs could be produced and exert some (residual) activity. To test this possibility, we used the OSMR-expressing HUH7 cell line in which STAT3 is phosphorylated (p-STAT3) upon OSM treatment (Figure 3C) (29). As expected, recombinant human OSM (rhOSM), used as positive control, induced p-STAT3 in HUH7, as inferred by the shift of p-STAT3 signal detected by fluorocytometry and the increase in mean fluorescence intensity (MFI) of p-STAT3 (Figure 3E). Supernatants from EPO-activated WT UT7, UT7^G8-WT^, and UT7^C3-WT^ cells consistently induced a net shift in p-STAT3 signal (Figure 3F) that translated into a significant increase in p-STAT3 MFI in the HUH7 cell line (Figure 3G), confirming the presence of active OSM in these supernatants. In contrast, the supernatant from EPO-activated UT7^C7-OSMfs^ cells demonstrated a negligible effect on the p-STAT3 status of HUH7 (Figure 3F). This was evidenced by a significantly lower p-STAT3 induction in comparison to that observed in WT settings (Figure 3G). This result suggests that the OSMfs protein, which lacks the FxxK motif necessary to bind OSMR, if produced, is unable to interact with and/or transduce a signal through the OSM receptor (30). Overall, our genetic, structural, and functional analyses suggested that the *OSM* variant identified in the patients corresponds to a bona fide LoF mutation.

### Osm signaling is required for embryonic RBC formation and neutrophil differentiation in zebrafish.

**Figure 4:**
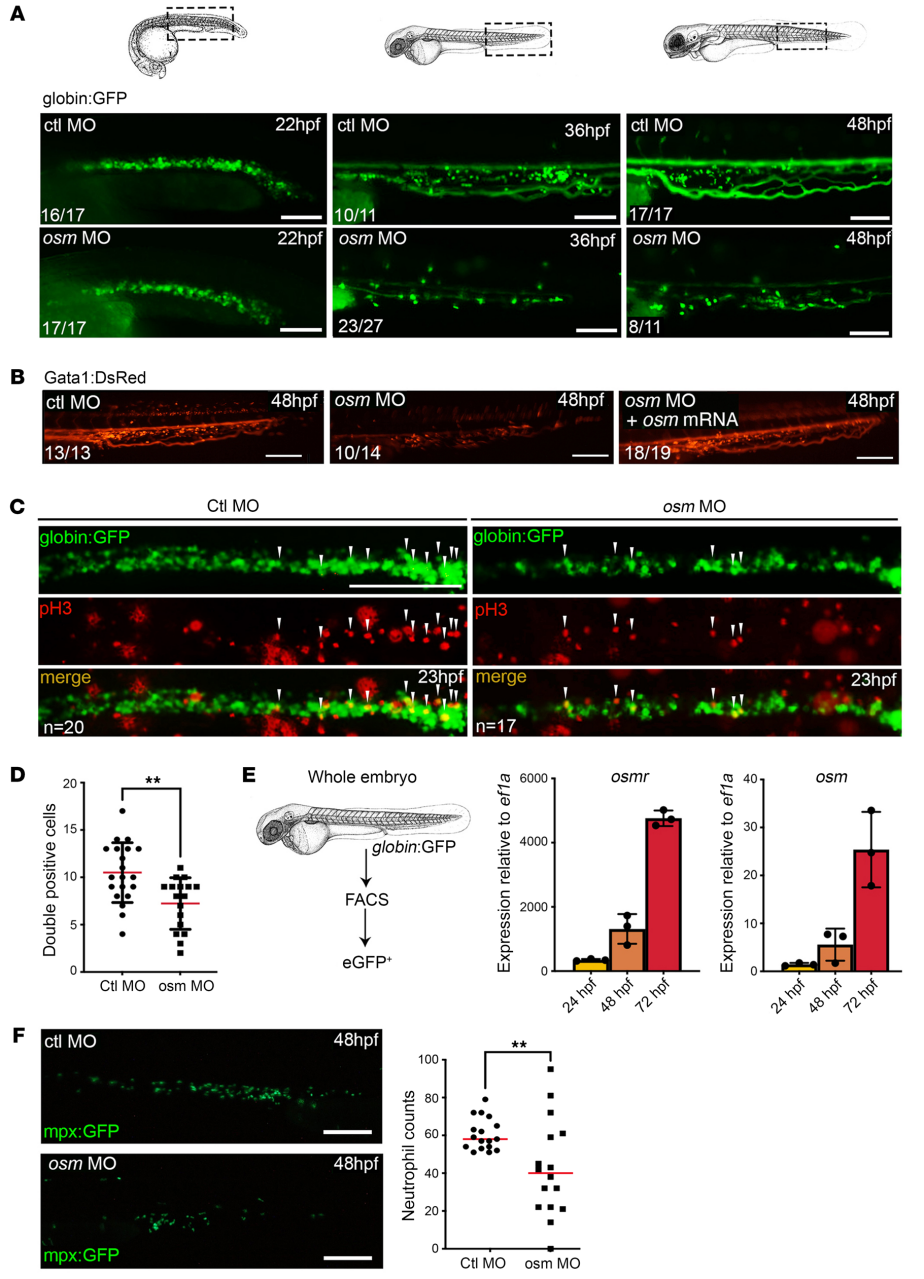
*Osm*-morphants show erythropoiesis defect. (**A**) *globin:GFP* embryos were injected with control- or *osm*- morpholino (MO), and observed by fluorescence microscopy to measure their content in erythrocytes. Dashed rectangles highlight the part of zebrafish in which the cells are detected. Numbers in bottom left of images indicate the number of embryos displaying the indicated phenotype out of the total number of embryos analyzed. Scale bars: 100 μm. (**B**) The strong reduction of circulating erythrocytes (detected by *gata1:DsRed* marker) in *osm*-MO is rescued by overexpressing *osm* (noted + *osm* mRNA). Numbers in bottom left of images indicate the number of embryos displaying the indicated phenotype out of the total number of embryos analyzed. Scale bars: 200 μm. (**C**) 23 hpf *globin:GFP* embryos were stained for phospho-histone 3 (pH3), a marker of proliferation. Arrows indicate colocalization of pH3 and globin:GFP. Scale bar: 100 μm. (**D**) Fewer *globin:GFP/pH3* double-positive cells are detected in *osm*-morphants, demonstrating a decrease in proliferating erythroid progenitors. Statistics derived from unpaired *t* tests with α = 0.05. **P < 0.01. (**E**) Quantitative PCR performed on sorted erythrocytes (GFP^+^ cells) at different stages show that they express high levels of *osmr* and *osm* transcripts. (**F**) Left: *mpx:GFP* embryos were injected with control- or *osm*- morpholino (MO) and observed by fluorescence microscopy to measure their content in neutrophils. Right: Fewer *mpx:GFP* cells are detected in *osm*-morphants, demonstrating a decrease in neutrophils. Statistics derived from unpaired *t* tests with α = 0.05. **P < 0.01.

Next, we wanted to investigate the role of the Osm/Osmr axis in the development of thrombocytes (the functional equivalent of mammalian platelets), RBCs, and neutrophils in zebrafish. We previously described the Osm-encoding gene in the zebrafish genome (*osm*, previously annotated as *si:ch73-47f2.1*) (31), while the *osmr* gene was already annotated. Our previous work on definitive hematopoiesis during zebrafish embryogenesis demonstrated a requirement of Osm/Osmr signaling in hematopoietic stem cell specification, at the level of the aortic hemogenic endothelium (31). To investigate the role of this signaling pathway in thrombocyte production, we injected a splice-morpholino targeting *osm* in *cd41:GFP* embryos and analyzed the *cd41:GFP* positive cells corresponding to thrombocytes. The number of thrombocytes in Osm-deficient animals was not significantly reduced as compared with WT animals, suggesting a minor role, if any, of the Osm/Osmr axis in the production of these cells in zebrafish (Supplemental Figure 8). Next, to assess the role of Osm/Osmr in RBC production, we injected the *osm* morpholino into 1-cell stage *globin:GFP* or *gata1:DsRed* embryos, in which all erythrocytes express GFP or DsRed, respectively. No difference between *osm*-morphants and controls could be observed at 22 hours post-fertilization (hpf), when primitive RBC progenitors are still in the intermediate cell mass (ICM), before they enter circulation (Figure 4A). Strikingly however, as early as 36 hpf, we observed a loss of circulating erythrocytes in the majority of tested Osm-deficient embryos (Figure 4A). Importantly, the defect in circulating erythrocytes was fully rescued by injection of *osm* full-length mRNA (Figure 4B). The loss of circulating erythrocytes was also found in *osmr* morphants (Supplemental Figure 9A), demonstrating that both ligand and receptor are important for erythropoiesis. Moreover, *gata1* and *cmyb* were highly decreased in the caudal hematopoietic tissue of *osmr*-morphants, showing that definitive hematopoiesis (including the development of definitive erythrocytes) was deeply impaired (Supplemental Figure 9B). This loss of erythropoiesis was not due to cell death (data not shown), but rather to a defect in erythroblast proliferation in the ICM, as scored by anti-phospho-histone 3 (pH3) staining in *globin:GFP* embryos at 23 hpf (Figure 4, C and D). Interestingly, quantitative PCR indicated that *osmr* was highly expressed by the erythroid lineage, which also expressed relatively high levels of *osm*. This suggests that these cells may have a potential autocrine and/or paracrine role (Figure 4E).

We next injected the *osm* morpholino in either *mpeg1.1:GFP* or *mpx:GFP* embryos, where macrophages and neutrophils are marked by GFP, respectively. While we could not observe any difference in the number of macrophages (Supplemental Figure 10), the number of neutrophils was significantly reduced (Figure 4F). Taken together, these results indicate that the Osm/Osmr axis participates in neutrophil differentiation and plays a critical role in erythrocyte production in zebrafish.

## Discussion

**Figure 5:**
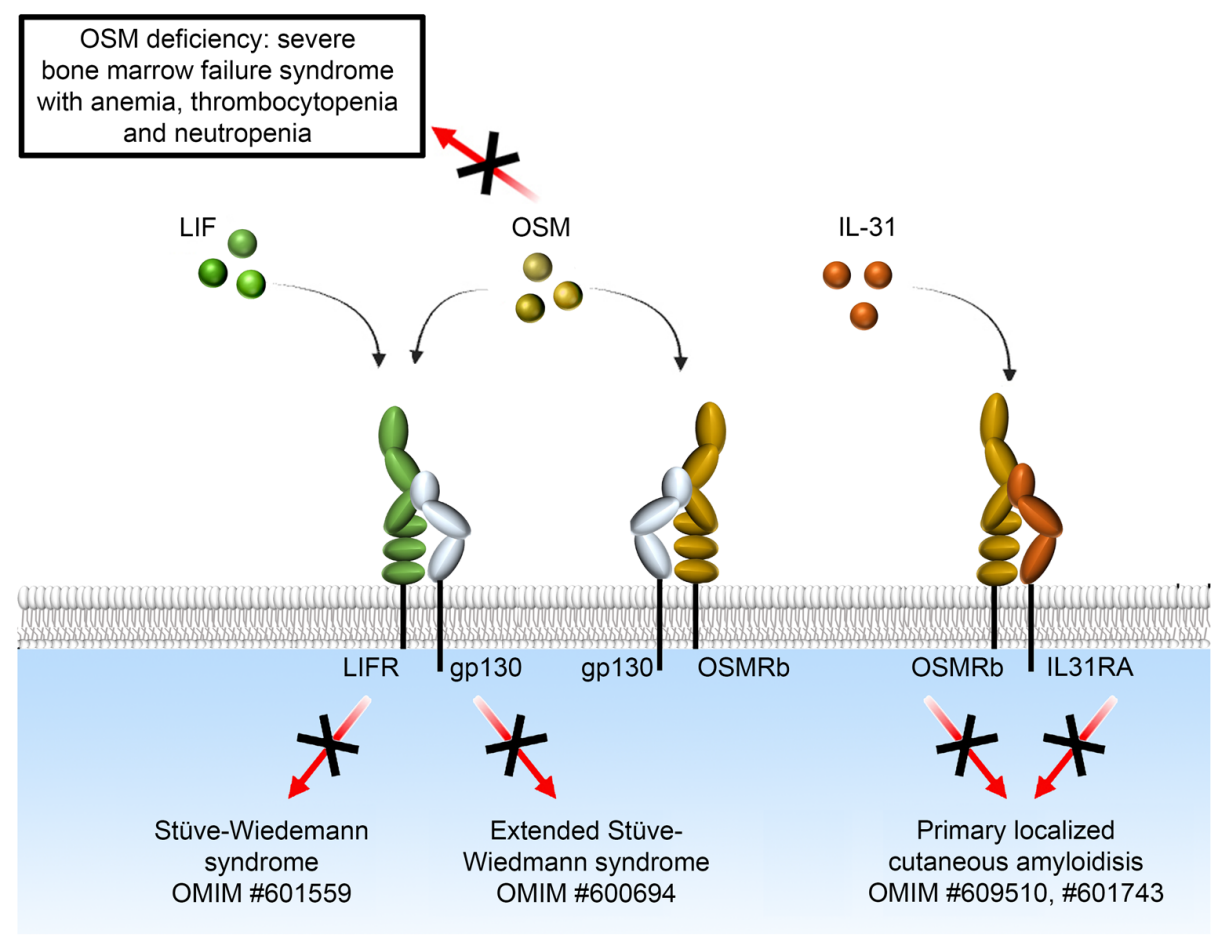
OSM deficiency as a novel inherited bone marrow failure syndrome. Schematic representation of the complexity of the signaling pathways of OSM and OSMRb and the diseases associated with their deficiency in humans. The OMIM number for each disease is shown.

Among the cytokines of the IL-6 family, OSM has the peculiarity of binding 2 different receptors associated with the common gp130 subunit: LIFR and OSMR (Figure 5). On the other hand, the OSMR subunit (OSMRb) is used in conjunction with the IL31RA protein to form the IL-31 receptor (Figure 5). Because of this complex network, our knowledge of the specific function of each of these factors (cytokines and receptor subunits) remains limited, especially in humans. However, the identification of human diseases associated with germline LoF mutations in some of the aforementioned factors has provided invaluable information to better understand their respective roles in vivo. For example, biallelic LoF mutations in the LIFR gene cause Stüve-Wiedemann syndrome-1 (STWS1; OMIM:601559), a fatal neonatal disorder characterized by skeletal dysplasia, feeding difficulties, respiratory distress, and hyperthermia (Figure 5) (32–35). Interestingly, biallelic LoF mutations in *IL6ST*, the gene encoding gp130, are associated with extended Stüve-Wiedemann syndrome (36) (OMIM:600694). Thus, although gp130 interacts with several cytokine receptor subunits (37), its deficiency mainly causes the clinical manifestations seen in LIFR deficiency, suggesting that most of the symptoms observed in gp130-deficient patients originate from an impaired LIF/LIFR signaling pathway during embryogenesis (Figure 5) (37). In the same line, individuals carrying a LoF mutation in either OSMRb (OMIM:601743) or IL-31RA (OMIM:609510) exhibit primary localized cutaneous amyloidosis (Figure 5) (18, 38). This supports the notion that the clinical manifestations observed in OSMRb deficiency are mainly due to defective IL-31 signaling. Thus, despite the description of these genetic diseases and a plethora of studies conducted in various cellular and animal models, the specific function of OSM in vivo has remained elusive, especially in humans.

In the present study, we report 3 individuals from a consanguineous family carrying a homozygous 1 base-pair insertion in the coding sequence of OSM. This insertion created a frameshift and replaced the C-terminal part of OSM, which contains the FxxK motif necessary for interaction with LIFR and OSMR, with a neopeptide. Our structural and functional analyses suggested that this OSM variant corresponds to a LoF mutation. The patients exhibited profound anemia, thrombocytopenia, and neutropenia while other blood lineages were unaffected. These clinical manifestations emphasized a predominant role for OSM in the differentiation of erythrocytes, platelets and, to a lesser extent, granulocytes, an observation consistent with the finding that healthy individuals injected with high doses of neutralizing anti-OSM antibodies exhibited mild anemia and thrombocytopenia (39). In addition, *Osm*-KO and *Osmr*-KO mice show a mild decrease in platelets and erythrocytes, further supporting a role for OSM in erythropoiesis and megakaryopoiesis. The observation that patient HSPCs differentiate normally into erythroblasts and thrombocytes in vitro (Figure 1 and Supplemental Table 2) indicates that OSM deficiency may either result in a cell-intrinsic defect in HSPCs that impedes their differentiation beyond the erythroblast and megakaryocyte stages and/or that it alters the bone marrow environment, thereby impairing hematopoiesis. Notably, experiments showing that transfer of bone marrow cells from WT mice to Osmr-deficient mice results in a decrease in erythrocytic/megakaryocytic precursors argue for a critical role of OSM/OSMR signaling in the bone marrow environment to maintain normal hematopoiesis rather than a cell-intrinsic defect (23, 40, 41).

Given the multiple functions that have been attributed to OSM, one of the most surprising findings of our study is the rather limited, albeit severe, clinical manifestations found in patients. In fact, apart from the hematopoietic defects, the patients do not show any developmental abnormalities, infectious diseases, or cognitive delays, although it cannot be excluded that other clinical manifestations may occur in a specific context (infection, bone injury, etc.). Furthermore, as we observed a progressive decrease in erythrocyte, platelet, and neutrophil levels in patients (Figure 1), it is conceivable that the symptoms could worsen over time and affect other blood lineages and/or organs, as observed in Osm-deficient mice in which the bone marrow environment deteriorates with age (40). Careful attention should be paid to ensure that the patients do not develop other diseases with age. As the administration of recombinant Osm has been shown to increase platelet counts in normal mice (42), it is reasonable to assume that the injection of recombinant hOSM into patients may have a hematopoietic benefit. However, to the best of our knowledge, clinical grade recombinant hOSM is not yet available.

Our zebrafish experiments used a morpholino approach to directly target the expression of either Osm or Osmr. As this strategy involves the injection of antisense morpholinos at the 1-cell stage immediately after fertilization, we were only able to study the potential involvement of Osm/Osmr during the first 4 days of zebrafish development, which corresponds to primitive hematopoiesis and the onset of definitive hematopoiesis for this model. We did not find any major defects in thrombopoiesis. The precise origin of the first thrombocytes is still unclear during zebrafish embryogenesis. Whether they share a common precursor with primitive RBCs (43) or arise from erythro-myeloid progenitors (44) has not been fully elucidated. In addition, although thrombocytes are the functional equivalent of mammalian platelets, they do not derive from a similar differentiation pathway as megakaryocytes (45), which appears to be an important differentiation stage affected by OSMR signaling in mammals (23, 42). It is therefore possible that zebrafish thrombopoiesis does not depend on Osm signaling, and a different approach using Osm or Osmr mutants would be more appropriate to test this hypothesis in adult animals. In contrast, although our model showed that the specification of primitive RBCs from the mesoderm was unaffected by deficient Osmr signaling, as the ICM harbored normal numbers of primitive RBC progenitors, we observed impaired proliferation at later stages, leading to a decrease in circulating RBCs as early as 36 hpf, which could be reversed by the overexpression of *osm* full-length mRNA. We also observed a significant decrease in neutrophils, but not macrophages, in OSM morphants, similar to what we observed in OSM-deficient patients (*P* < 0.01). We can therefore conclude that the role of the Osm/Osmr pathway in erythropoiesis and granulopoiesis appears to be conserved from zebrafish to mammals.

In conclusion, we have identified homozygous OSM LoF mutations associated with IBMFS, which is characterized by profound anemia, thrombocytopenia, and neutropenia. Having observed that OSM-deficient HSPCs normally differentiate into erythroblasts and megakaryocytes in vitro, we speculate that human OSM deficiency in patients alters the bone marrow microenvironment to compromise RBC, platelet, and neutrophil production. Further efforts are needed not only to identify other pathogenic *OSM* variants that may expand the phenotypes associated with OSM deficiency, but also to better understand the function of OSM in human hematopoiesis.

## Methods

### Sex as a biological variable

The 3 OSM-deficient individuals described in this study are females. It is impossible to discriminate zebrafish embryos sexually, so obviously, sex was not considered as a biological variable.

### Cell preparation

Bone marrow mononuclear cells were isolated by density gradient separation on lymphocyte separation medium (Lymphoprep, Eurobio). CD34^+^ hematopoietic progenitors were isolated using an indirect CD34^+^ microbead kit and a separator (VarioMACS; Miltenyi Biotec), according to the manufacturer’s instructions. Purified CD34^+^ cells were stained with anti-CD34–allophycocyanin (APC; clone 581; BD Pharmingen) and the purity of the CD34^+^ cells was checked with a MACSQuant analyzer (Miltenyi Biotec).

### In vitro liquid erythroid differentiation

Erythroid differentiation potential was assessed by liquid culture. CD34^+^ HSPCs isolated from bone marrow were cultured in IMDM supplemented with 15% BIT (Stem Cell), 100 ng/mL of human recombinant stem cell factor, 10 ng/mL IL-3, and 100 ng/mL IL-6 proteins (R&D Systems). The medium was changed on day 3 and the IL-6 was replaced by EPO (2 U/mL) (epoetin α; Eprex). After 7 days, cells were harvested and analyzed by flow cytometry using a combination of FITC-conjugated anti-huCD71 (clone M-A712), phycoerythrin-conjugated (PE-conjugated) anti-huCD45 (clone HI30), allophycocyanin-conjugated (APC-conjugated) anti-huCD235a (Glycophorin a) (clone HIR2), and VioBlue-conjugated anti-huCD36 antibodies (the anti-CD71, anti-CD45, and anti-CD235a were provided by BD Pharmingen, and the anti-CD36 was provided by Miltenyi Biotec). Analysis was performed on a FACS MACSQuant Analyser using Kaluza software.

### CFU assay

Erythroid and granulocyte/monocyte differentiation potential was assessed by culture in a semisolid medium. CD34^+^ HSPCs from bone marrow were plated at a concentration of 1 × 10^3^ cells/mL in a methylcellulose-based medium (Methocult H4435 enriched, Stem Cell Technologies), which is formulated to support optimal growth of erythroid progenitor and granulocyte-macrophage progenitor cells. Cells were cultured for 10 days in 6-well plates at 37°C in humidified 5% CO_2_. CFU-GM and BFU-E colonies were counted after 10 days and classified according to the morphology of the colony using an inverted microscope.

### MegaCult CFU-Mk assay

MegaCult-C serum-free media (Stem Cell Technologies) have been formulated to promote the growth of human colony-forming unit– megakaryocyte (CFU-Mk) in collagen-based medium. It contains all the reagents needed to stain fixed and dehydrated CFU-Mk–derived colonies cultured in MegaCultTM-C collagen–based medium. Megakaryocytes expressing the glycoprotein IIb/IIIa were stained with anti-human CD41 antibody and an alkaline phosphatase detection system. CD34^+^ HSPCs from bone marrow were added at a concentration of 5 × 10^4^ cells/mL per chamber (in 0.75 mL of medium) of a double-chamber slide for 12 days of culture. At day 12, the slides were dehydrated, fixed, and stained for anti-human CD41 antibody according to the manufacturer’s protocols. All colonies were microscopically classified into 3 categories: megakaryocytic colony (MK), mixed MK colony (mixed), no MK colony (no MK) and scored using an inverted microscope. Results were also expressed as total colonies, which included pure, mixed, and no MK colonies.

### Purification of blood monocytes and activation

Monocytes were purified from PBMNCs using positive selection (Kit BD IMag Anti-Human CD14 Magnetic Particles, BD Biosciences). The monocytes were stimulated upon Toll-like receptor (TLR) with 100 ng/mL of LPS (InvivoGen) for 3 days in RPMI 1640-GlutaMAX supplemented with 10% fetal bovine serum and 1% penicillin-streptomycin. After 3 days, the supernatants were collected and OSM and IL-6 production were assessed by ELISA.

### CRISPR/Cas9-mediated mutagenesis

The sequence 5′-AGACACGGCTGAGCCCACGA-3′ located in the *OSM* sequence was used as gRNA and cloned into the pSpCas9(BB)-2A-GFP vector (pX458; a gift from Feng Zhang [MIT, Boston, Massachusetts, USA]; Addgene plasmid 48138). UT7 cells were transfected with both the pX458-OSM vector and 5 μg of a 140 nucleotide-long DNA matrix containing the mutated OSM sequence (5′-C*A*GGAACAACATCTACTGCATGGCCCAGCTGCTGGACAACTCAGACACGGCTGAGCCCACGAAGGCTGGgCCGGGGGGCCTCTCAGCCGCCCA CCCCCACCCCTGCCTCGGATGCTTTTCAGCGCAAGCTGGAGGGCT*G*C-3′; IDT), where asterisks correspond to phosphorothioate-modified bases and the g in lower case to the insertion. The cells were cultured in the presence of 10 μM of Nedisertib (DNA-PKcs inhibitor). Seventy-two hours after transfection, GFP-positive UT7 cells were cell sorted and cloned in 96-well plates. Clones were assessed by Sanger sequencing for the presence of the *OSM* insertion. Two unmutated UT7 cell clones (C3 and G8) were selected and used as WT clones.

### Cell culture and treatment

We used the UT7/EPO line whose cells show more marked erythroid differentiation and are strictly dependent on EPO. This line is derived from the UT7 cells (46). Cell line UT7/EPO was a gift from G. Courtois (Imagine Institute, Paris, France). UT7/EPO cells were cultured at 37°C with 5% CO_2_ α-MEM (Gibco, Thermo Fisher Scientific) supplemented with 10% fetal bovine serum and 1% penicillin-streptomycin + 50 μg/mL gentamicin. Cell line HuH7 was a gift from S. Vaulont (Cochin Institute, Paris, France). HUH7 cells were cultured at 37°C with 5% CO_2_ in DMEM (Gibco, Thermo Fisher Scientific) supplemented with 10% fetal bovine serum and 1% penicillin-streptomycin + 50 μg/mL gentamicin.

### Cell stimulation

UT7/EPO cells (250 × 10^3^ cells/mL) were plated in 6-well dishes in α-MEM (Gibco, Thermo Fisher Scientific) supplemented with 10% fetal bovine serum and 1% penicillin-streptomycin + 50 μg/mL gentamicin + 1 U/mL EPO or minus EPO (Binocrit) during 72 hours. After 72 hours, the supernatants were collected and stored at –80°C. HUH7 cells were plated in 6-well dishes (1.5 × 10^6^ cells/well). Twenty-four hours later, the supernatant was removed and the HUH7 cells were incubated with the UT7 supernatants for 30 minutes. Cells were then collected and analyzed for intracellular phospho-Stat3 staining.

### Flow cytometry phospho-STAT3

Cells were harvested with 1× trypsin solution and fixed by adding an equal volume of Phosflow Fix Buffer I (BD Biosciences) for 10 minutes at 37°C and then permeabilized by adding cold Phosflow Fix Buffer III (BD Biosciences) for 30 minutes on ice. Cells were stained with Alexa Fluor 488 Mouse Anti-Phospho Stat3 (pY705) (BD Biosciences, clone 4/p-STAT3, 1:5) for 1 hour at room temperature protected from light. Flow cytometry analysis was performed on a Gallios Beckman Coulter flow cytometer. Results were analyzed using Kaluza software, version 2.2, and statistical analysis completed using unpaired Student’s *t* test in Microsoft Excel, version 16.92, or GraphPad Prism, version 10.4.1.

### Protein measurements

The production of OSM and IL-6 was measured in the supernatant by ELISA using commercial kits and following the manufacturer’s instructions (R&D Systems [DY206] for IL-6; Abcam [ab215543] for OSM).

### Genetic analysis

The DNA mutation numbering system used is based on cDNA sequence. Nucleotide numbering reflects cDNA numbering with 1 corresponding to the A of the ATG translation initiation codon in the reference sequence. The initiation codon is codon 1.

### WGHM

Genotyping with the Illumina Human Linkage-24 BeadChip was carried out according to standard methodology. In brief, each sample (200 ng) was whole genome amplified, fragmented, precipitated, and resuspended in an appropriate hybridization buffer. Denatured samples were hybridized on prepared Human Linkage-24 BeadChips for a minimum of 16 hours at 48°C. After hybridization, the BeadChips were processed for the single-base extension reaction, as well as staining and imaging on an Iscan (Illumina) and an Autoloader 2 (Illumina). Normalized bead intensity data obtained for each sample were loaded into the Illumina GenomeStudio software, version 2011.1, which converted fluorescence intensities into single nucleotide polymorphism genotypes.

### WES

Standard manufacturer protocols were used to perform target capture with the Illumina TruSeq Exome Enrichment Kit and sequencing of 100 bp paired-end reads on Illumina HiSeq. Approximately 10 Gb of sequence was generated for each subject, such that 90% of the coding bases of the exome defined by the consensus coding sequence project were covered by at least 10 reads. Adaptor sequences were removed, and quality trimmed reads were created with the Fastx toolkit (https://github.com/agordon/fastx_toolkit). Then a custom script was used to ensure that only read pairs with both mates present were subsequently used. Reads were aligned to hg19 with BWA31, and duplicate reads were marked with Picard (https://broadinstitute.github.io/picard/) and excluded from downstream analyses. Single nucleotide variants and short insertions and deletions were called by using SAMtools (http://samtools.sourceforge.net/) pileup and varFilter32 with the base alignment quality adjustment disabled and were then quality filtered to require at least 20% of reads supporting the variant call (47).

### 3D structure modeling and analysis

3D structure models of the OSM:OSMR:gp130 complex were built using ColabFold, version 1.5.5 (48). 3D structure models of OSMfs protein were built using AlphaFold3. AlphaFold3 was used for neopeptide modeling, as the importance of coevolutionary information (extracted from multiple sequence alignment [MSAs]) was considerably reduced compared with AlphaFold2. 3D structure visualization was performed with Chimera (49). Hydrophobic Cluster Analysis (HCA) plots of the amino acid sequences were drawn using the DrawHCA server at http://osbornite.impmc.upmc.fr/hca/hca-form.html

### Methods for zebrafish experiments

#### Zebrafish strains and husbandry.

AB* (WT), along with transgenic and mutant strains were kept in a 14-hour light/10-hour dark cycle at 28°C (50). We used the following transgenic animals: « Tg*(gata1:DRed)^sd2^* (51), Tg*(cd41:eGFP)^la21l^* (45), Tg*(globin:eGFP)^cz3325^* (52), Tg*(mpeg1.1:GFP)^gl22^* (53), and Tg*(mpx:GFP)^i113^* (54). Hereafter, transgenic lines will be referred to without the Tg(XXX:XXX) nomenclature.

#### Morpholinos and mRNA injections.

All morpholinos oligonucleotides (MOs) were splice-blocking MOs and were purchased from Gene Tools. MO efficiency was previously tested by PCR (31). The MO sequences are listed in Supplemental Table 4. The *osm* mRNA has been described before (31) and was injected at 300 ng per embryo.

#### Whole-mount in situ hybridization staining and analysis.

Whole-mount in situ hybridization (WISH) was performed on 4% paraformaldehyde-fixed (PFA-fixed) embryos at the developmental time points indicated. Digoxygenin-labeled probes were synthesized using an RNA labeling kit (SP6/T7; Roche). RNA probes were generated by linearization of TOPO-TA or ZeroBlunt vectors (Invitrogen) containing the PCR-amplified cDNA sequence. WISH was performed as previously described (55). Phenotypes were scored by comparing expressions with siblings. All injections were repeated 3 independent times. Analysis was performed using GraphPad Prism software, version 10.4.1. Embryos were imaged in 100% glycerol using an Olympus MVX10 microscope.

#### Immunofluorescence staining.

Immunofluorescence double staining was carried out and analyzed as described previously (56), with chicken anti-GFP (1:400, Life Technologies, A10262) and rabbit anti-phospho-histone 3 (pH3) antibodies (1:250, Abcam, ab5176). Alexa Fluor 488–conjugated anti-chicken secondary antibody (1:1000, Life Technologies, A11039) and Alexa Fluor 594–conjugated anti-rabbit secondary antibody (1:1000, Life Technologies, A11012) were used to reveal primary antibodies.

#### Imaging.

Images were obtained with a Nikon SMZ1500 microscope and Z stacks were made through the entire embryo. All images were processed using cell sense dimension software and Adobe Photoshop, version 21.0. To score for myeloid differentiation, embryos were imaged with a MVX10 stereoscope (Olympus).

#### Cell sorting and flow cytometry.

Zebrafish transgenic embryos (15 to 20 per biological replicate) were incubated in 0.5 mg/mL liberase (Roche) solution and shaken for 90 minutes at 33°C, then dissociated, filtered, and resuspended in 0.9× PBS and 1% FCS. Dead cells were labeled and excluded by staining with 5 nM SYTOX red (Life Technologies) or 300 nM DRAQ7 (Biostatus). Cell sorting was performed using an Aria II (BD Biosciences) or a Bio-Rad S3 (Bio-Rad). Data were analyzed using FlowJo, version 10.10.0, and statistical analysis completed using unpaired Student’s *t* test in Microsoft Excel, version 16.92, or GraphPad Prism, version 10.4.1.

#### qPCR and qPCR analysis.

For zebrafish analysis, RNA was extracted using the QIAGEN RNeasy Mini Kit (Qiagen) and reverse transcribed into cDNA using a Superscript III Kit (Invitrogen) or qScript (Quanta Biosciences). Quantitative real-time PCR (qPCR) was performed using KAPA SYBR FAST Universal qPCR Kit (KAPA BIOSYSTEMS) and run on a CFX connect Real-Time System (Bio Rad). All primers are listed in Supplemental Table 5. Analysis was performed using an unpaired Student’s *t* test in Microsoft Excel or GraphPad Prism.

### Statistics

Statistical analyses were performed on Prism (GraphPad Software). Student’s *t* test (2 tailed, type 2) was used to determine significant differences. *P* < 0.05 was considered significant.

### Study approval

Blood and bone marrow samples were collected from the individuals after the provision of written, informed consent. Genetic studies and data collection procedures were approved by the local institutional review board (Comité de Protection des Personnes Ile de France II, Paris, France and the French Advisory Committee on Data Processing in Medical Research [Comité Consultatif sur le Traitement de l’Information en matière de Recherche dans le domaine de la Santé, Paris, France]). According to Swiss and European legislation, there is no requirement for any approval concerning experiments performed on zebrafish aged less than 5 days after fertilization.

### Data availability

Any information as well as Supporting data values are available from the author upon request. Values for all data points in graphs are reported in the Supporting Data Values file.

## Author contributions

AG participated in the design of the study of the functional impact of the OSM mutation and performed most of the corresponding experimental work. AG analyzed the results and prepared figures and tables. LK, SS, IF, CBM, HS, M Chouteau, and SL performed some experiments. CLP designed and set up some experiments and supervised part of the experiments. PR performed genetic analysis and identified *OSM* variants. M Cavazzana and ES supervised part of the project. MCLBK and AA performed and supervised some experiments. VCD assisted with some clinical studies. MW, AS, and JR identified the affected patients and assisted with related clinical and laboratory studies. IC performed structural analysis. JYB and CBM performed experiments in zebrafish models. JBS performed and analyzed the histological data. CLP, IA, and PR conceived and supervised the project. PR, CLP, JYB, and IA wrote the manuscript with editing contributions from all authors.

## Supplementary Material

Supplemental data

Supporting data values

## Figures and Tables

**Table 1:**
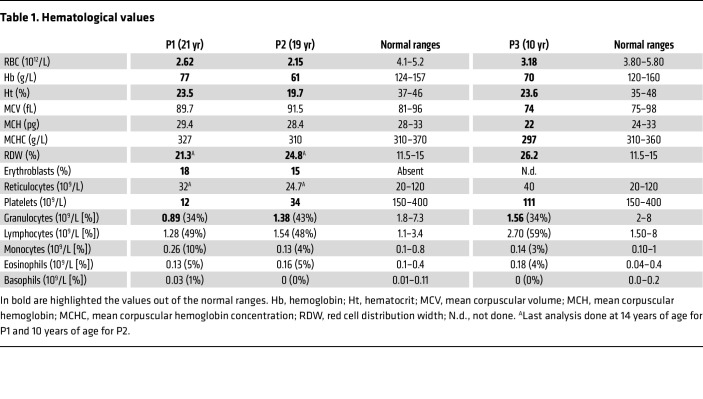
Hematological values

## Version 1 01/23/2025

In-Press Preview

## Version 2 03/17/2025

Electronic publication
