# Supplementary material for: Human oncostatin M deficiency underlies an inherited severe bone marrow failure syndrome
Source: J Clin Invest. 2025 Jan 23;135(6):e180981. doi: 10.1172/JCI180981 (PMC11910226; doi:10.1172/JCI180981)
Supplement: Supplemental data [file jci-135-180981-s025.pdf]

Human Oncostatin M deficiency underlies an inherited severe bone marrow failure syndrome

Supplementary Figure 1

A

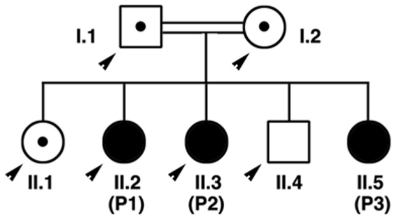

B

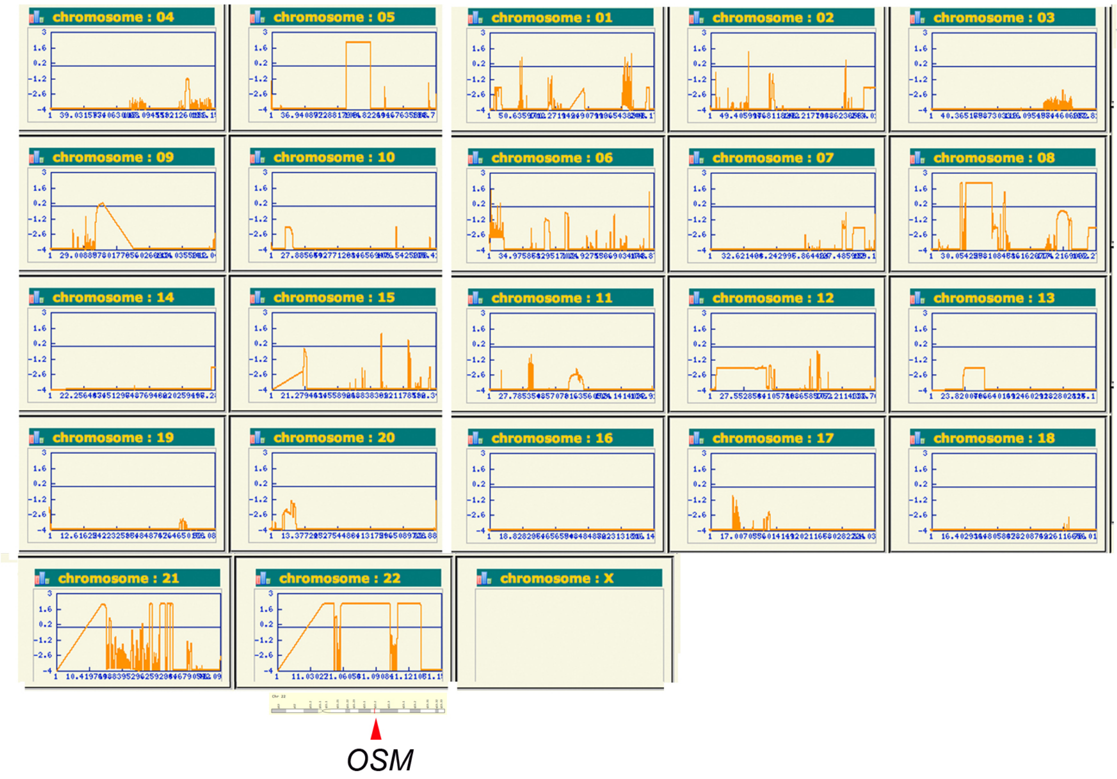

Supplementary Figure 1. Whole genome homozygosity mapping (WGHM) analysis. (A) pedigree of the family. Black arrows point to the individuals involved in the WGHM analysis. **(B)** Representation of the logarithm of the odds (LOD) scores obtained by WGHM analysis according to the chromosomal position. Orange parts that are above the blue horizontal lines correspond to chromosomal regions found homozygous only in patients P1 and P2 (P3 was not analyzed). The position of the *OSM* gene located in chromosome 22 is highlighted by a red arrow.

Supplementary Figure 2

A

***FBXL17*; c.328G>A; p.E110K**

Features	Values	Descriptions
gnomAD exome:	0	v2.1.1 Exomes **global MAF**
gnomAD genome:	No match in gnomAD genome	v2.1.1 Genomes **global MAF**
gnomAD exome (non cancer):	.	v2.1.1 Exomes **MAX MAF** for non-cancer dataset
gnomAD v4 Genome:	No match in gnomADv4 Genome	v4.0.0 Genomes **global MAF**
gnomAD v4 Exome:	5.545e-06	v4.0.0 Exomes **global MAF**
dbSNP rsid:	rs1187208655	Identifier for NCBI dbSNP
Clinvar Germline:	No match in Clinvar v20240421	Clinvar interpretation
ClinGen EvRepo:	This variant has not yet been assessed by the ClinGen experts groups.	ClinGen Evidence Repository Classification for the variant
hg38 InterVar:	Uncertain significance with the following criteria: PM2	Semi-automated ACMG classification - click on the intervar link to adjust - passing the mouse over a criterion will display the definition
GeneBe:	Uncertain significance as no criterion could be applied	Semi-automated ACMG classification - click on the GeneBe link to adjust - passing the mouse over a criterion will display the definition
LOVD Matches:	No match in LOVD public instances	LOVD match in public instances

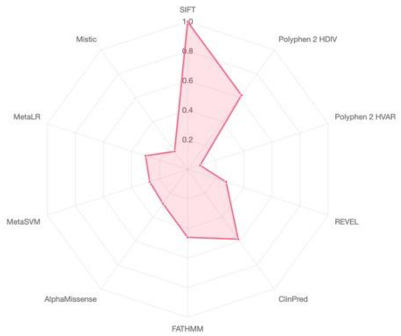

B

***DGKG*; c.14G>A; p.R5Q**

Features	Values	Descriptions
gnomAD exome:	4.376e-05	v2.1.1 Exomes **global MAF**
gnomAD genome:	3.185e-05	v2.1.1 Genomes **global MAF**
gnomAD exome (non cancer):	6.711e-05	v2.1.1 Exomes **MAX MAF** for non-cancer dataset
gnomAD v4 Genome:	3.94e-05	v4.0.0 Genomes **global MAF**
gnomAD v4 Exome:	8.14e-05	v4.0.0 Exomes **global MAF**
dbSNP rsid:	rs578252543	Identifier for NCBI dbSNP
Clinvar Germline:	Uncertain_significance ★ ☆ ☆ ☆	Clinvar interpretation
ClinGen EvRepo:	This variant has not yet been assessed by the ClinGen experts groups.	ClinGen Evidence Repository Classification for the variant
hg38 InterVar:	Uncertain significance with the following criteria: PM1 BP4	Semi-automated ACMG classification - click on the intervar link to adjust - passing the mouse over a criterion will display the definition
GeneBe:	Likely benign with the following criteria: PM2 BP4_Strong	Semi-automated ACMG classification - click on the GeneBe link to adjust - passing the mouse over a criterion will display the definition
LOVD Matches:	No match in LOVD public instances	LOVD match in public instances

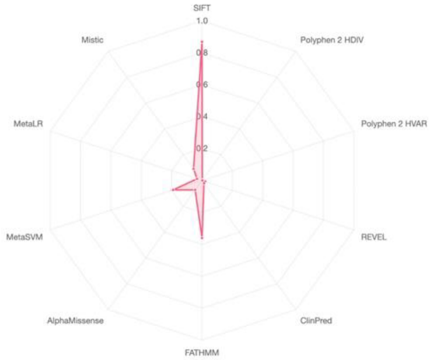

Supplementary Figure 2. Analysis of the variants identified in *FBLX17* (A) and *DGKD* (B) genes. (Left) Representation of the variants in the various databases and prediction of their significance. (Right) Radar chart representing the prediction of the deleterious impact of variants as reported by different tools in Mobidetails (Baux D et al., Eur J Hum Genet. 2021).

Supplementary Figure 3

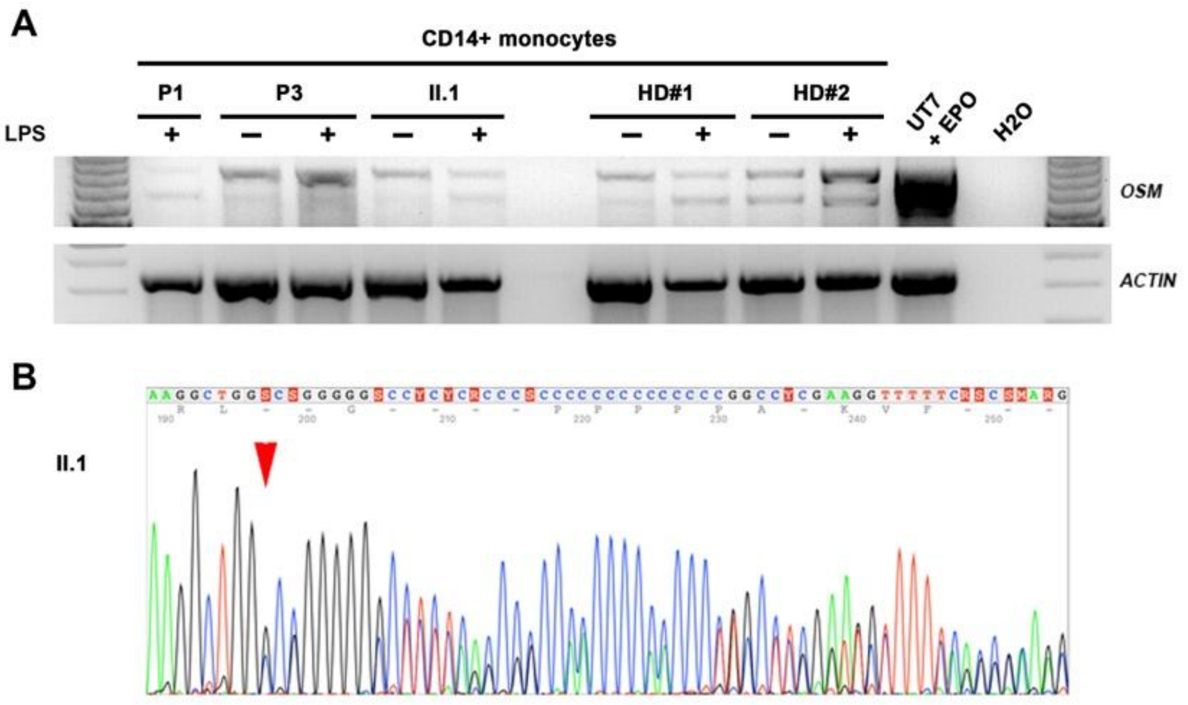

Supplementary Figure 3. Analysis of *OSM* transcript in patients' cells. (**A**) RT-PCR amplification of the full length OSM transcript in monocytes from two healthy donors (HD#1 and HD#2), the patients P1 and P3, and the healthy sister II.1 activated or not with lipopolysaccharide (LPS). cDNA from UT7 activated by EPO are used as a positive control. The similar patterns suggest that the mutation does not affect OSM expression and splicing. (**B**) The similar peak heights corresponding to the wild type and mutated *OSM* sequences obtained by Sanger sequencing from the healthy sister II.1 carrying the *OSM* insertion at a heterozygous state indicates that the transcript carrying the insertion is not subjected to degradation or destabilization.

Supplementary Figure 4

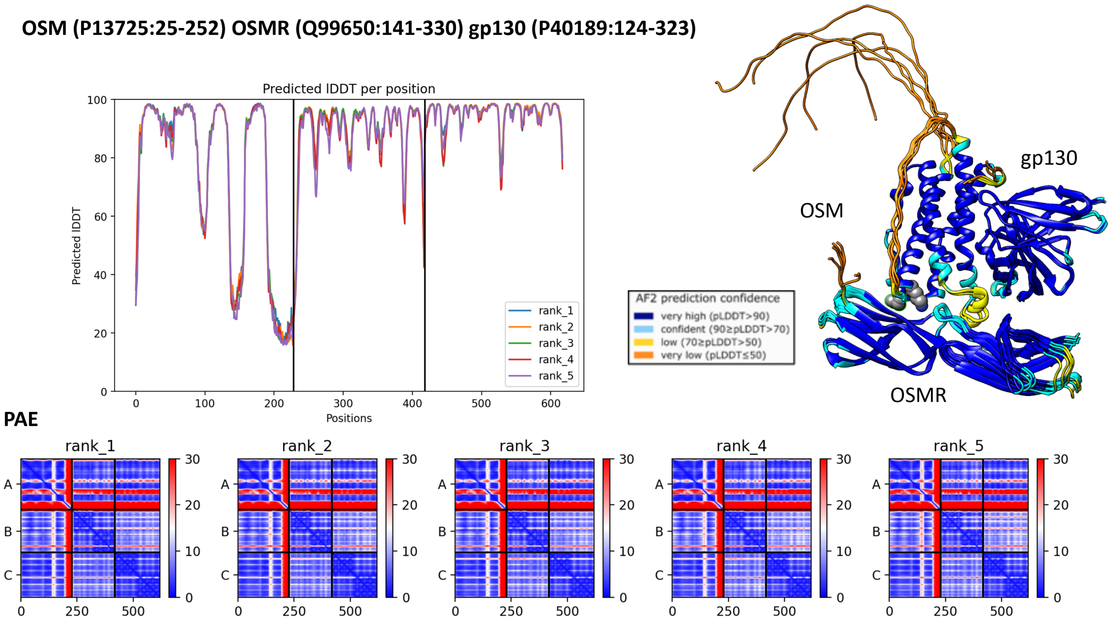

Supplementary Figure 4: Alphafold2 3D structure models of the human OSM:OSMR:gp130 complex. Five similar 3D structure models were generated using ColabFold v1.5.5 (1). The superimposition of these models, colored according to the pLDDT values (predicted Local Dstance Difference Test, corresponding to a per amino acid confidence measure), are shown at right. PAE (Predicted Aligned Error) maps are given at bottom, giving a measure of the confidence in the relative position of two residues within the predicted structure.

Supplementary Figure 5

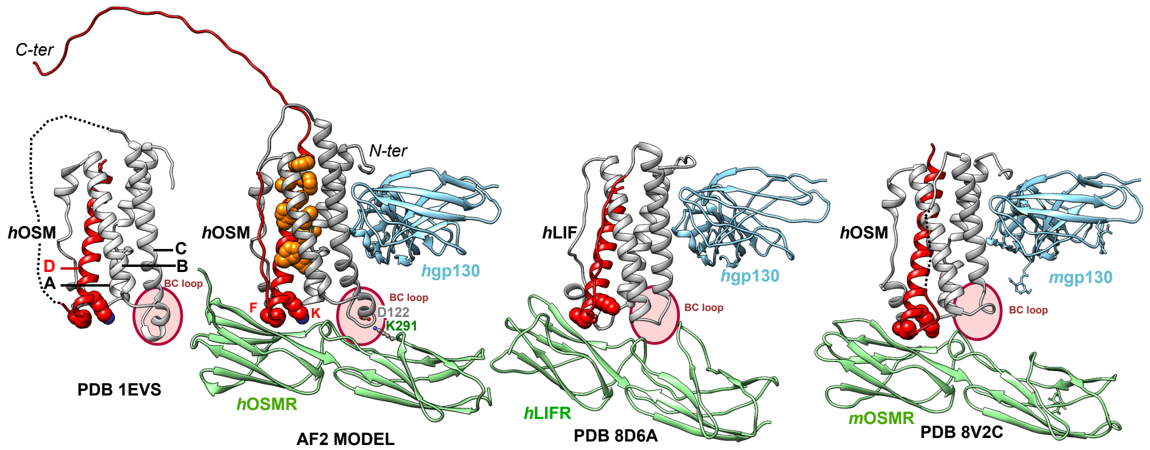

Supplementary Figure 5: Comparison of the AlphaFold2 model of human *(h)* OSM:OSMR:gp130 complex with experimental 3D structures. At left is shown the experimental 3D structure of human *(h)* OSM (pdb:1EVS,(2)), and at right are shown those of the human *(h)* LIF:LIFR:gp130 complex (pdb:8D6A, (3)) and the mouse *(m)* OSM:OSMR:gp130 complex (pdb:8V2C, (4)). The large CD loop (dashed line) was unsolved in the experimental 3D structure of isolated *h*OSM, which is otherwise similar to the AlphaFold2 model of *h*OSM (1.5 Å on 163 superimposed C-alpha atoms). Only small variations can be observed in the conformation of the BC loop, in contact in the complex with *h*OSMR (salt-bridge between *h*OSM D122 and *h*OSMR K291). The AlphaFold2 model of the *h*OSM:*h*OSMR:*h*gp130 closely resembles the experimental 3D structure of the *h*LIF:*h*LIFR:*h*gp130 complex (18 % and 32 % of sequence identity with OSM and OSMR, respectively), except from the conformation of the BC loop, which is shorter in this last case. This BC loop has been shown to be critical for determining the relative affinity for OSMR-OSM-gp130 and LIFR-OSM-gp130 (5). A shorter BC loop is also observed in the experimental 3D structure of the *m*OSM:*m*OSMR:*m*gp130 complex (solved after AF2 model building), while the overall 3D structure of this complex is also similar to the AF2 model of the *h*OSM: *h*OSMR: *h*gp130 complex.

Supplementary Figure 6

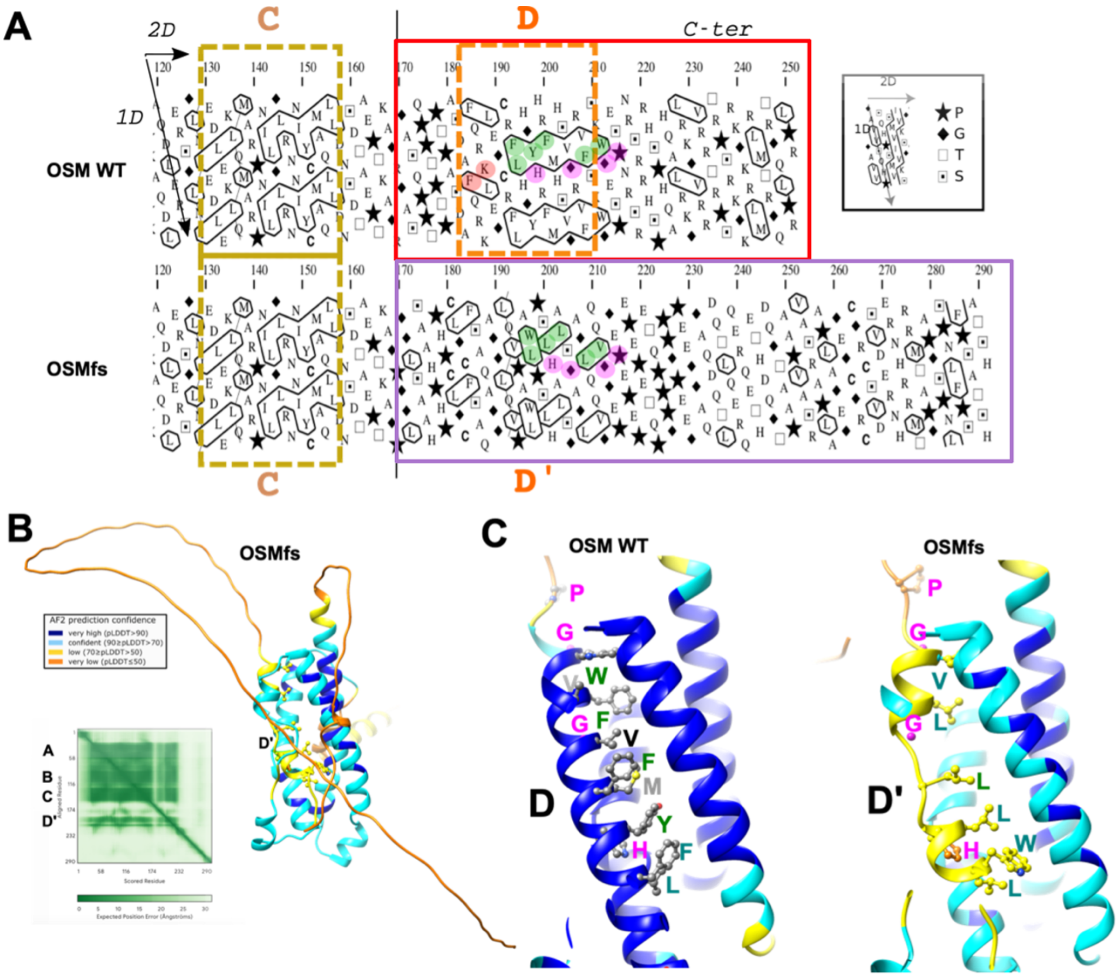

Supplementary Figure 6. Structural analysis of the OSMfs protein. (A) HCA plots comparing the C-terminal part of the WT (top) and mutated form (OSMfs-bottom) of OSM. Hydrophobic cluster analysis (HCA) is a two-dimensional representation of the protein sequence, in which strong hydrophobic amino acids (V,I,L,F,M,Y,W) are highlighted. These form clusters, which mainly correspond to regular secondary structures. Such a representation allows to distinguish regions which are folded (dense in hydrophobic clusters) from those which are disordered (containing few and small hydrophobic clusters (6). The way to read the sequence (1D) and secondary structures (2D) is indicated in the inset. The position of the helices of the four-helix bundle (pdb:1EVS) are indicated with dashed boxes (C and D). In the red box is highlighted the WT OSM part that is missing in the mutated OSM. The neopeptide generated by the frameshift (c.507_508insG; p.Arg170Alafs*124) is highlighted in the purple box. This sequence appeared globally disordered, like the very C-terminal end of the WT OSM (aa 213-252). However, several hydrophobic amino acids grouped into clusters (green) can be aligned at the level of OSM helix D, suggesting a possible similarity between the two sequences. Other conserved positions are shown in pink. **(B) AlphaFold3 3D structure model of OSMfs.** Five similar 3D structure models were produced, one of which is shown here, colored according to the pLDDT values (*predicted Local Distance Difference Test, corresponding to a per amino acid confidence measure*). High PAE values (*Predicted Aligned*

Error, giving a measure of the confidence in the relative position of two residues within the predicted structure) are observed for the sequence segment which can be aligned with WT OSM helix D (hence named D'). **(C) Comparison of the 3D structure models of WT OSM and OSMfs at the level of OSM helix D.** The amino acids aligned in panel A (green – hydrophobic, pink - others) are shown in atomic details, occupying similar positions in the 3D structure, oriented towards the core of the protein. Even though the pLDDT values are low for the D' segment, such a correspondence suggests that it may mimic OSM helix D and allow the protein to fold.

Supplementary Figure 7

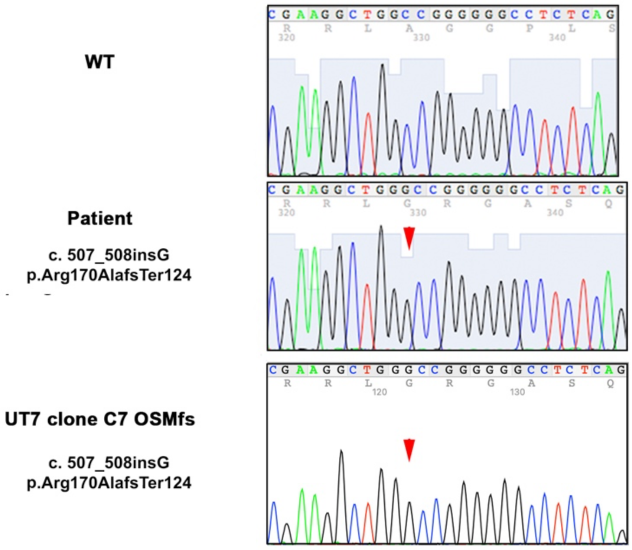

Supplementary Figure 7. Sequencing analysis of the *OSM* mutation in the UT7 clone C7. Sanger sequencing of the *OSM* one base pair insertion detected in the patient and in the CRISPR/Cas9-modified UT7 clone C7.

Supplementary Figure 8

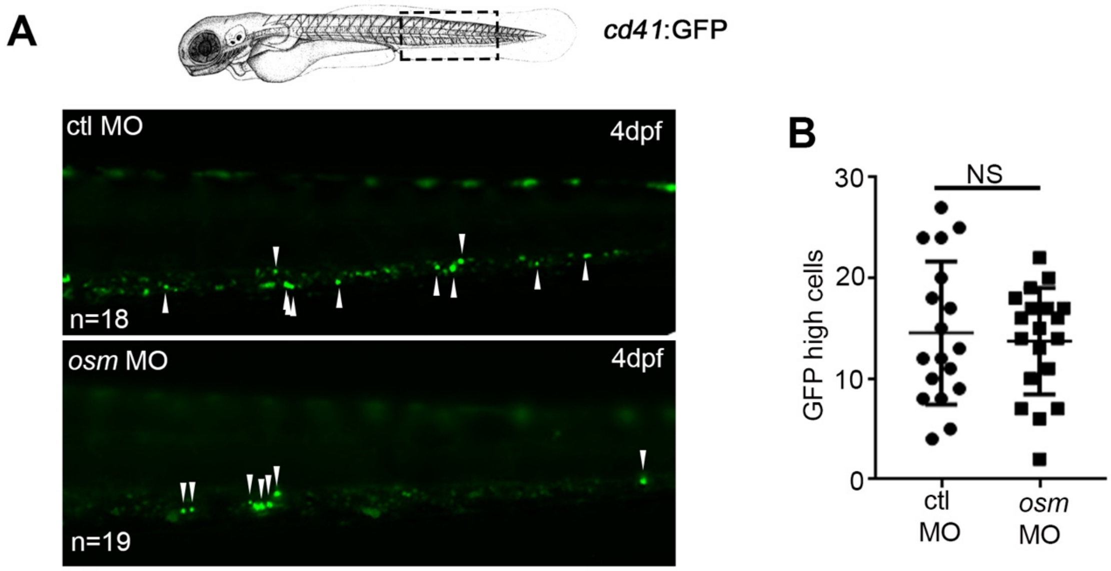

Supplementary Figure 8. Thrombocyte detection in *cd41:GFP* zebrafish (A) Thrombocytes corresponded to GFP high cells in the *cd41:GFP* zebrafishes. **(B)** No significant differences were found in zebrafish at 4 days post fertilization (dpf) injected with a control morpholino (ctl MO) and with the *osm* morpholino (*osm* MO).

Supplementary Figure 9

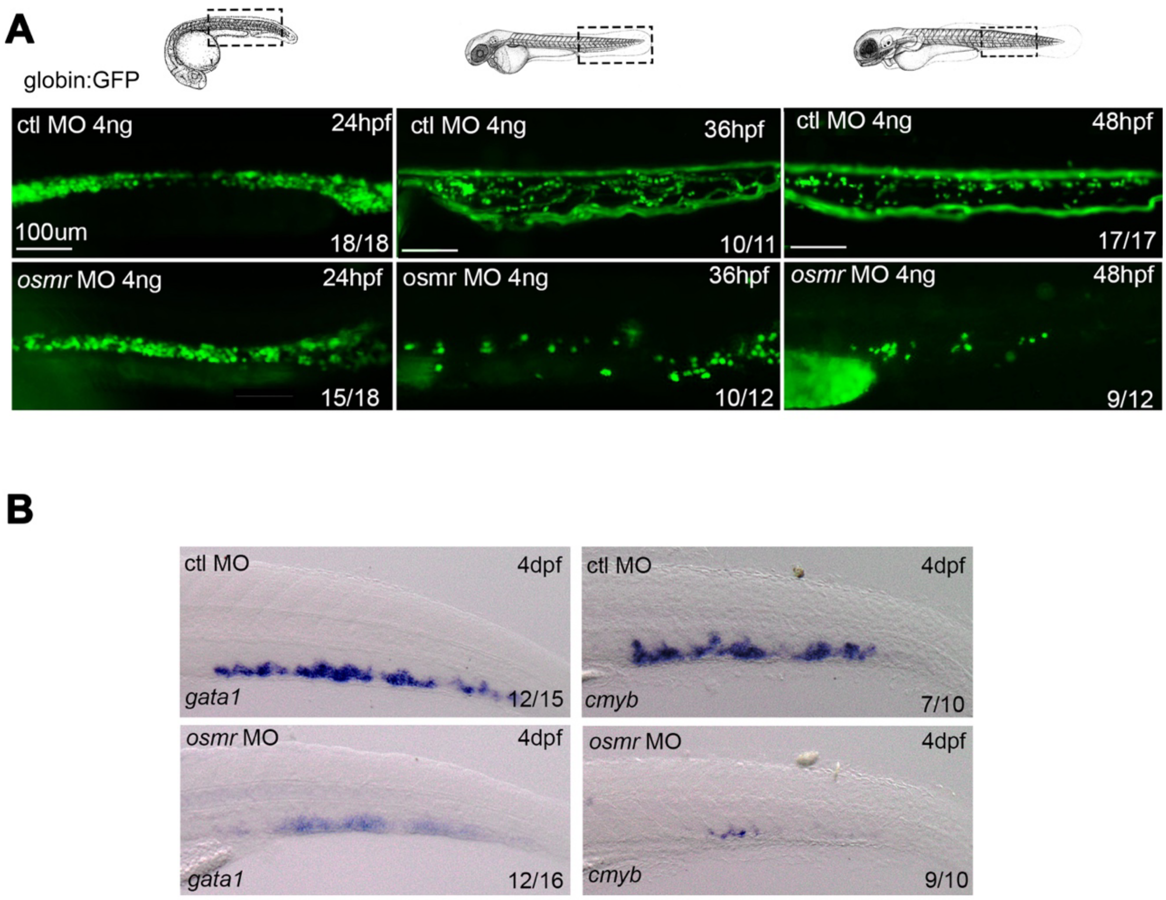

Supplementary Figure 9. *osmr*-morphants show erythropoiesis defects. (A). *globin:GFP* embryos were injected with control- or *osmr*- morpholino (MO), and observed by fluorescence microscopy to measure their content in erythrocytes. Dashed rectangles highlight the part of zebrafish in which the cells are detected. Numbers in bottom right of images indicate the number of embryos displaying the indicated phenotype out of the total number of embryos analyzed. Scale bar: 100µm. (**B**) Control-MO and *osmr*-MO injected embryos were scored for the expression of *gata1* and *cmyb* at 4dpf. Numbers in bottom right of images indicate the number of embryos displaying the indicated phenotype out of the total number of embryos analyzed.

Supplementary Figure 10

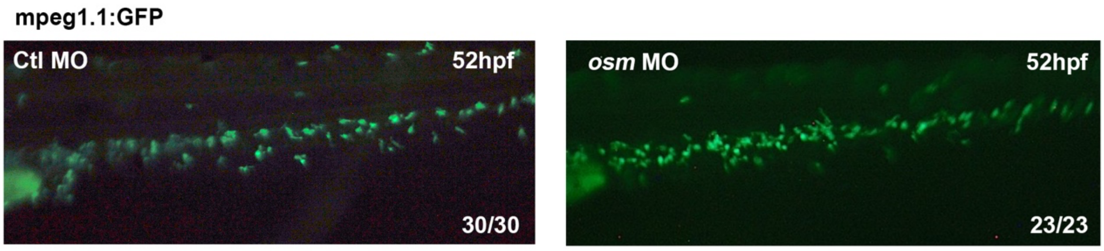

Supplementary Figure 10. ***osm*-morphants do not show defect in macrophage production defects.** *Mpeg1.1:GFP* embryos were injected with control- or *osm*- morpholino (MO), and observed by fluorescence microscopy to measure their content in macrophages.

Supplementary Table 1: Bone marrow cellularity

	P1					P2	HD
	3 yrs	8 yrs	9.5 yrs	14 yrs	18 yrs	7 yrs	
Cellular density*	**high**	n.d.	n.d.	normal	**low**	n.d.	normal
Lymphoid lineage (%)	**22**	**36**	14	**40**	n.d.	**28**	5-15
Granulocytic lineage (%)	**23**	**38**	57	**39**	n.d.	**46**	50-75
Erythroid lineage (%)	**52**	22	22	12	n.d.	24	8-30
Megakaryocytes	**rare**	**rare**	**rare**	**absent**	n.d.	**absent**	present
Myeloid/erythroid ratio	**0.4**	**1.7**	2.6	3.3	n.d.	**1.9**	2.5-6

**cellular density estimated on bone marrow biopsies.*
n.d. not done. HD: healthy donors.
Bold text shows out-of-range values.

Supplementary Table 2. Colony-forming cell assay

**/10^3 BM CD34$^+$**	**Exp. #1**		**Exp. #2**			**Normal ranges**
	P3	Mother	P1	P2	Control	
CFU-GM	21	19	8	6	5	7-101
BFU-E	6	4	55	53	73	1-74

**/10^5 BM CD34$^+$**	**P1**	**P2**	**Control**
Colony number	25	62	18
CFU-MK (%)	52	43.5	50
Mixed (%)	20	21	11.1
Non MK (%)	28	35.5	38.9

*BM: bone marrow; CFU-GM: colony forming unit of granulocytes and macrophages; BFU-E: burst-forming unit-erythroid; CFU-MK: colony forming unit of megakaryocyte*s

Supplementary Table 3

Features	Values	Descriptions
LOVD Matches:	No match in LOVD public instances	*LOVD match in public instances*
gnomAD v4 Genome:	No match in gnomADv4 Genome	*v4.0.0 Genomes **global MAF***
gnomAD v4 Exome:	No match in gnomADv4 Exome	*v4.0.0 Exomes **global MAF***
gnomAD genome:	No match in gnomAD genome	*v2.1.1 Genomes **global MAF***
gnomAD exome:	No match in gnomAD exome	*v2.1.1 Exomes **global MAF***
gnomAD exome (non cancer):	No match in gnomAD exome	*v2.1.1 Exomes **MAX MAF** for non-cancer dataset*
GeneBe:	Uncertain significance with the following criteria: PM2	*Semi-automated ACMG classification - click on the GeneBe link to adjust - passing the mouse over a criterion will display the definition*
dbSNP rsid:	No match in dbSNP v156	*Identifier for NCBI dbSNP*
Clinvar Germline:	No match in Clinvar v20240421	*Clinvar interpretation*
ClinGen EvRepo:	This variant has not yet been assessed by the ClinGen experts groups.	*ClinGen Evidence Repository Classification for the variant*

Supplementary table 3. *Absence of detection of the OSM variant* c.507_508insG, p.Arg170AlafsTer124 in databases as listed in Mobidetails (7)

Supplementary Table 4. Morpholinos used in the study

Morpholino	Sequence	Concentration used
Control MO	CCTCCTACCTCAGTTACAATTTATA	7ng
osm MO	AGCACTAAAGCCTAATACTTACGT	7ng
osmr MO1	CCTTTAATGTGAGGAATCACCTGTA	4ng

Supplementary Table 5. qPCR primers used in the study

Ef1a F	GAGAAGTTCGAGAAGGAAGC
Ef1a R	CGTAGTATTTGCTGGTCTCG
Osm F	AAACCCCTCATTTCTAAGACCA
Osm R	GTTCTTCAAGTCAAGTTCAGGA
Osmr F	TGGACAGCACAGCAGCTC
Osmr R	CAGCTGCGGGTCACTGC

Supplementary References

1. Mirdita M, Schutze K, Moriwaki Y, Heo L, Ovchinnikov S, and Steinegger M. ColabFold: making protein folding accessible to all. *Nat Methods.* 2022;19(6):679-82.
2. Deller MC, Hudson KR, Ikemizu S, Bravo J, Jones EY, and Heath JK. Crystal structure and functional dissection of the cytostatic cytokine oncostatin M. *Structure.* 2000;8(8):863-74.
3. Zhou Y, Stevis PE, Cao J, Saotome K, Wu J, Glatman Zaretsky A, et al. Structural insights into the assembly of gp130 family cytokine signaling complexes. *Science advances.* 2023;9(11):eade4395.
4. Zhou Y, Stevis PE, Cao J, Ehrlich G, Jones J, Rafique A, et al. Structures of complete extracellular assemblies of type I and type II Oncostatin M receptor complexes. *Nat Commun.* 2024;15(1):9776.
5. Chollangi S, Mather T, Rodgers KK, and Ash JD. A unique loop structure in oncostatin M determines binding affinity toward oncostatin M receptor and leukemia inhibitory factor receptor. *The Journal of biological chemistry.* 2012;287(39):32848-59.
6. Bitard-Feildel T, Lamiable A, Mornon JP, and Callebaut I. Order in Disorder as Observed by the "Hydrophobic Cluster Analysis" of Protein Sequences. *Proteomics.* 2018;18(21-22):e1800054.
7. Baux D, Van Goethem C, Ardouin O, Guignard T, Bergougnoux A, Koenig M, et al. MobiDetails: online DNA variants interpretation. *Eur J Hum Genet.* 2021;29(2):356-60.
